# Cardiovascular Computed Tomography in the Diagnosis of Cardiovascular Disease: Beyond Lumen Assessment

**DOI:** 10.3390/jcdd11010022

**Published:** 2024-01-12

**Authors:** Zhonghua Sun, Jenna Silberstein, Mauro Vaccarezza

**Affiliations:** 1 Curtin Medical School, Curtin University, Perth, WA 6102, Australia; jenna.beinart@student.curtin.edu.au (J.S.); mauro.vaccarezza@curtin.edu.au (M.V.); 2 Curtin Health Innovation Research Institute (CHIRI), Curtin University, Perth, WA 6102, Australia

**Keywords:** cardiac computed tomography, 3D, visualization, diagnosis, coronary artery disease, 3D printing, virtual reality, mixed reality, artificial intelligence

## Abstract

Cardiovascular CT is being widely used in the diagnosis of cardiovascular disease due to the rapid technological advancements in CT scanning techniques. These advancements include the development of multi-slice CT, from early generation to the latest models, which has the capability of acquiring images with high spatial and temporal resolution. The recent emergence of photon-counting CT has further enhanced CT performance in clinical applications, providing improved spatial and contrast resolution. CT-derived fractional flow reserve is superior to standard CT-based anatomical assessment for the detection of lesion-specific myocardial ischemia. CT-derived 3D-printed patient-specific models are also superior to standard CT, offering advantages in terms of educational value, surgical planning, and the simulation of cardiovascular disease treatment, as well as enhancing doctor–patient communication. Three-dimensional visualization tools including virtual reality, augmented reality, and mixed reality are further advancing the clinical value of cardiovascular CT in cardiovascular disease. With the widespread use of artificial intelligence, machine learning, and deep learning in cardiovascular disease, the diagnostic performance of cardiovascular CT has significantly improved, with promising results being presented in terms of both disease diagnosis and prediction. This review article provides an overview of the applications of cardiovascular CT, covering its performance from the perspective of its diagnostic value based on traditional lumen assessment to the identification of vulnerable lesions for the prediction of disease outcomes with the use of these advanced technologies. The limitations and future prospects of these technologies are also discussed.

## 1. Introduction

Computed tomography (CT) is a widely used imaging modality in the diagnosis of cardiovascular diseases [1,2,3,4,5,6,7,8,9,10,11,12,13,14]. The diagnostic value of cardiovascular CT has been significantly enhanced with the rapid advancements in CT technologies over the last few decades, allowing for the acquisition of high-resolution images with low radiation doses [15,16,17,18,19]. In addition to the routine use of single-energy CT in daily practice, dual-source and dual-energy CT are becoming increasingly available in most of the current multi-slice CT scanners, further enhancing the diagnostic value of using cardiovascular CT in the context of many cardiovascular diseases [15,16,17,18,19,20,21].

The recent emergence of photon-counting CT (PCCT) represents the latest technological development of CT scanning techniques, with this technique having superior advantages over traditional CT scanners. PCCT enables the acquisition of high-resolution images with improved contrast resolution and simultaneous multi-energy imaging, showing superior advantages over traditional dual-energy CT in cardiovascular imaging [22,23,24,25,26,27,28,29].

Although cardiovascular CT is continuously gaining widespread acceptance and significance, its clinical value mainly focuses more on lumen assessment and the detection of vascular abnormalities, and this feature meets the diagnostic requirements in most situations due to its high diagnostic accuracy, thus serving as the first-line imaging modality in cardiovascular disease [1,2,3,4,5,6,7,8,9,10,11,12,13,14,15,16,17,18,19,20,21]. The well-known limitation of cardiovascular CT lies in the fact that it does not yield functional information, which is more apparent in the assessment of lesion-specific ischemia in coronary artery disease. This has been overcome with the increasing use of CT-derived fractional flow reserve (FFRCT) [30,31,32,33,34]. Single- and multi-center studies have confirmed that FFRCT can guide patient treatment by identifying lesion-specific ischemia, with coronary CT angiography (CTA) showing improved specificity and positive predictive values when compared to conventional coronary CTA in the diagnosis of coronary artery disease [35,36,37,38,39,40,41,42,43,44,45,46,47,48].

CT-derived post-processing approaches have transformed the clinical value of cardiovascular CT in cardiovascular disease, and this transformation has led to the creation of patient-specific physical models such as 3D-printed personalized heart and vascular models [49,50,51,52,53,54,55,56,57,58,59,60,61,62,63] and virtual models for visualization, created using virtual reality (VR), augmented reality (AR), and mixed reality (MR) [64,65,66,67,68]. These innovative 3D visualization tools augment the current applications of cardiovascular CT to go beyond the traditional diagnostic approach, as these novel tools can be used for educational purposes, medical training, and the simulation of cardiac procedures, as well as to enhance doctor–patient communication [49,50,51,52,53,54,55,56,57,58,59,60,61,62,63,64,65,66,67,68]. Further, with the increasing use of artificial intelligence (AI) in medicine, the role of cardiovascular CT has also been enhanced with machine learning (ML) and deep learning (DL) algorithms being incorporated into standard practices to improve the workflow of current practices, in addition to increasing diagnostic accuracy and the prediction of disease outcomes [69,70,71,72,73].

This review article provides an overview of cardiovascular CT in cardiovascular disease, with a focus on its current applications beyond lumen assessment. This includes describing the diagnostic value of using the latest CT scanners; PCCT; CT-derived 3D printing; VR, AR, and MR; FFRCT; and AI, ML, and DL in cardiovascular applications. Limitations and future research directions are highlighted. This review aims to provide a useful resource for readers who are interested in cardiovascular imaging research and the recent developments in CT technologies and their associated applications in cardiovascular disease.

## 2. Cardiovascular CT: Diagnostic Value Based on Standard Imaging Approach

Cardiovascular CT serves as the first-line imaging modality, being preferred over the gold standard conventional catheter-based angiography in the diagnosis of many cardiovascular diseases, including coronary artery disease (CAD), aortic aneurysms or dissection, peripheral artery disease, and pulmonary embolisms [1,2,3,4,5,6,7,8,9]. This is mainly because of its high diagnostic sensitivity and specificity (>90%) in most of these areas, hence proving that cardiovascular CT is a reliable alternative to conventional angiography. The main driving force of the technological developments in CT lies in cardiac imaging, in particular, the field of coronary CTA in CAD, which comes with strong demands in terms of both spatial resolution to detect and assess small coronary arteries and temporal resolution to allow for the acquisition of cardiac images with few motion-related artifacts [5,6,7,8,9,10]. Recent evidence has highlighted the high sensitivity (>90%) and very high negative predictive value (>98%) of coronary CTA in CAD, indicating that it is a reliable diagnostic tool for determining coronary stenosis. However, it is also a well-known fact that coronary CTA struggles to accurately assess heavily calcified plaques or coronary stented lumens due to blooming artifacts resulting from the extensive calcification and stent wires. This significantly hinders the diagnostic performance of coronary CTA, in particular, compromising its specificity and positive predictive value (PPV), limiting its widespread application in these two particular areas [74,75,76,77,78].

Various approaches have been proposed to address the issues encountered in coronary plaques, such as the use of image post-processing methods, iterative reconstruction, and thin slice thicknesses, as well as the use of AI algorithms [79,80,81,82,83,84,85,86,87,88,89]. This has improved coronary CTA performance to some extent; however, the specificity and PPV are still moderate (less than 70%) [88,89,90,91,92,93]. The recent advancements in CT technology and specifically the emergence of photon-counting CT have overcome this limitation to a greater extent, with superior spatial resolution and multi-energy imaging characteristics contributing to improving the diagnostic value [21,22,23,24,25,26,27,28]. 

## 3. Photon-Counting CT: The Latest Technological Advancements in Cardiovascular CT

Photon-counting detectors have the potential to overcome the current CT limitations by directly converting x-ray photons into electric signals, thus optimizing CT dose efficiency at an ultra-high resolution of 0.2 mm. The direct detection of photons also enables photons to be separated into specific energy levels, thus eliminating noise with an improved contrast-to-noise ratio. Further, PCCT allows for the acquisition of multi-energy images simultaneously; hence, PCCT has many benefits in cardiovascular imaging, specifically in assessing calcified coronary plaques or coronary stents with a reduction in noise and artifacts [21,22]. PCCT was introduced into clinical applications about two years ago, but increasing evidence derived from single- and multi-center studies has demonstrated its superior advantages over the current CT scanners, regardless of the presence of severe calcification in the coronary arteries or coronary stents [23,24,27,94,95,96].

**Figure 1:**
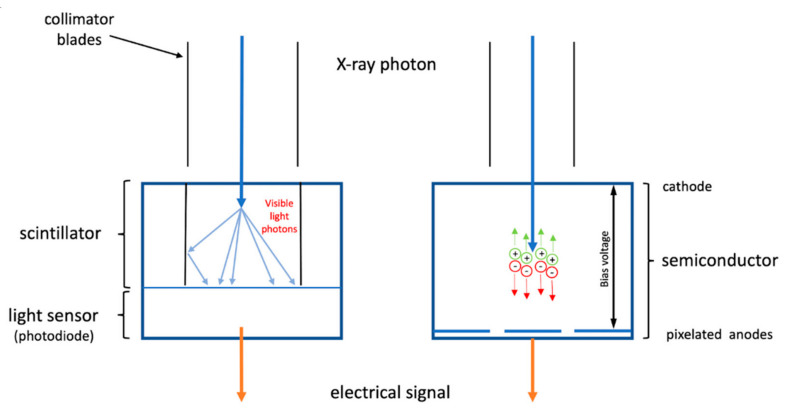
Imaging principle differences between standard energy-integrating detectors (EIDs) and photon-counting detectors (PCDs). X-ray photons are directly converted into electrical signals by a semiconductor on the PCD (**right image**); in contrast, X-ray photons are absorbed by the scintillator and converted into visible light, which is then collected by the light sensor that generates an electrical signal (**left image**). The long blue arrows indicate the difference of converting X-ray photons into electric signals between PCCT and EID CT. Reprinted with permission under open access from Tortora et al. [21].

**Figure 2:**
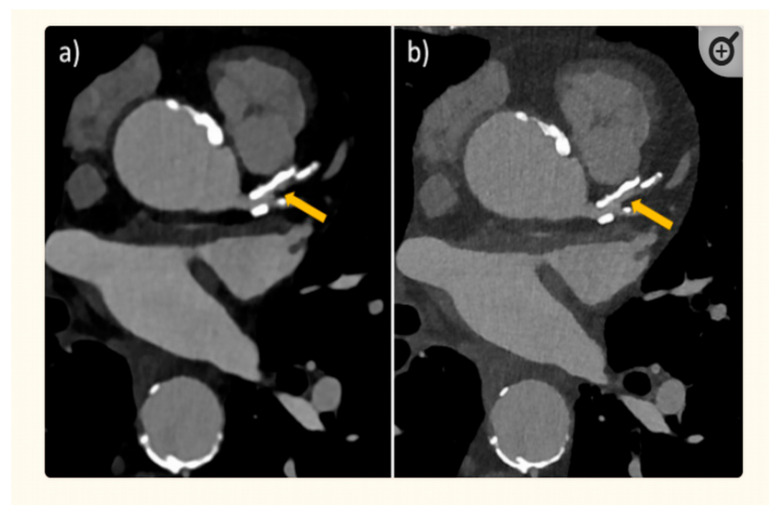
An 82-year-old man with coronary artery disease. The visualization of calcified plaques and the lumen diameter of the proximal left anterior descending coronary artery was improved via the acquisition of high-resolution photon-counting CT images (**b**) with 0.2 mm slice thickness rather than images obtained using standard CT (**a**) with 0.6 mm slice thickness. Arrow refers to larger perfused diameter of the proximal left anterior descending coronary artery in PCCT (**b**) than that observed with standard CT (**a**) which shows nearly occluded coronary lumen. Reprinted with permission under open access from Flohr et al. [24].

**Figure 3:**
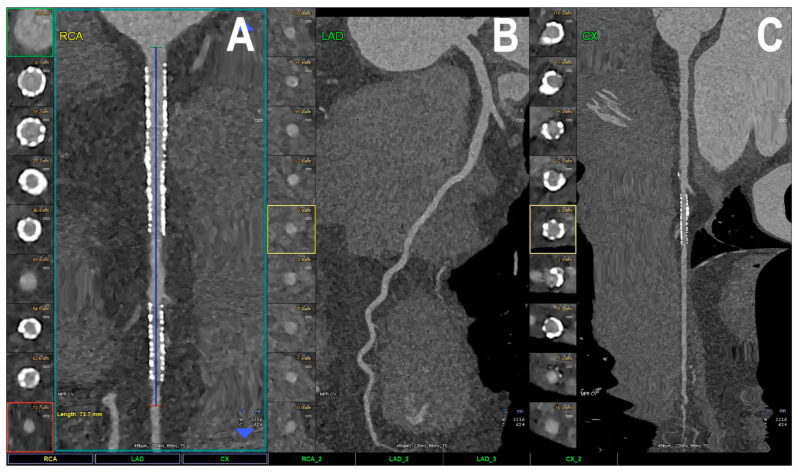
Cardiac PCCT visualization of coronary stents and stented lumen. There are two stents at the level of the proximal and middle RCA (**A**) and one stent on the marginal branch of the left LCx (**C**); the LAD (**B**) is normal, without any detectable atherosclerotic disease. All stents are perfectly visualized in terms of their inner struts and also in their inner lumen, which is difficult to achieve using standard cardiac CT. PCCT—photon-counting CT, LAD—left anterior descending, LCx—left circumflex, RCA—right coronary artery. Reprinted with permission under open access from Cademartiri et al. [22].

**Figure 4:**
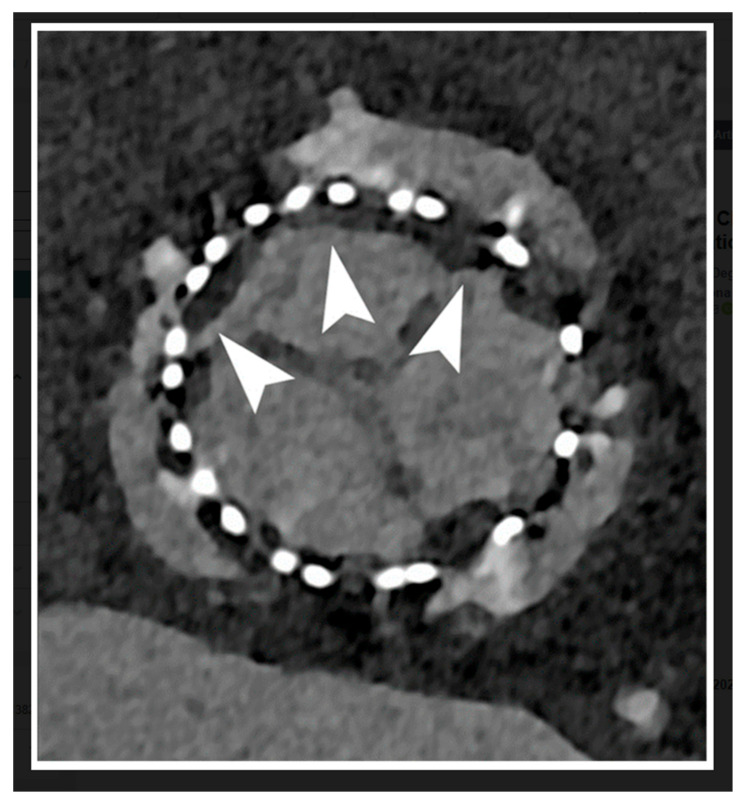
Cardiac PCCT: example of a follow-up of an aortic valve prosthesis that shows significant signs of Hypo-Attenuating Leaflet Thickening (HALT) due to thrombotic apposition (arrowheads). A very thin layer of hypodense tissue can be easily seen in the high-resolution PCCT image. Reprinted with permission under open access from Cademartiri et al. [22].

Figure 1 is a schematic representation of a comparison between PCCT and the current widely used CT scanners. Figure 2 shows an example of using PCCT in patients with high calcification in the coronary artery, with PCCT allowing for an improved visualization of the coronary lumen when compared to standard coronary CT angiography. Figure 3 shows a clear visualization of coronary stents and a stented lumen, while Figure 4 presents aortic valve prosthesis with the use of PCCT. Table 1 summarizes the main benefits of PCCT in cardiovascular applications [22].

## 4. Cardiovascular CT: Beyond Lumen Assessment

Despite providing excellent anatomical information about coronary arteries and other cardiovascular systems, the functional assessment of cardiovascular disease using CT is not comparable to that based on cardiac magnetic resonance imaging or nuclear medicine modalities. CT-derived image post-processing and analysis has overcome this limitation and has further advanced its clinical applications well beyond original lumen assessment. This is demonstrated by many innovative applications, including three-dimensional (3D) printed models based on CT data; VR, AR, and MR visualizations; and CT-derived FFR (FFRCT). The following sections provide an overview of the approaches involving the use of original cardiovascular CT images for post-processing and analysis and a summary of how these approaches enhance the clinical value of CT in cardiovascular disease.

### 4.1. Patient-Specific 3D-Printed Models: Medical Education

Three-dimensional printing has become an increasingly useful technology in medical applications, helping to generate patient-specific or personalized models to replicate normal anatomy and pathology with high accuracy. Studies based on randomized controlled trials have provided evidence that confirms the educational value of 3D-printed models in cardiovascular anatomy and pathology when compared to the current teaching methods [63,97,98,99]. The use of 3D-printed models has significantly increased students’ knowledge and understanding of complex cardiovascular anatomy and pathology, mainly in congenital heart disease (CHD), compared to the use of diagrams, cadavers, or lectures (the main techniques currently used in teaching) [97,98,99]. However, these randomized controlled studies have some limitations, either due to their small sample sizes, their singular focus on less complex cardiac anatomy without providing insight into the surrounding soft tissue structures [98], or their comparisons of 3D-printed models with virtual visualizations [100,101]. This latter limitation was addressed by a recent study that compared 3D-printed models with plastinated human specimens [102].

**Figure 5:**
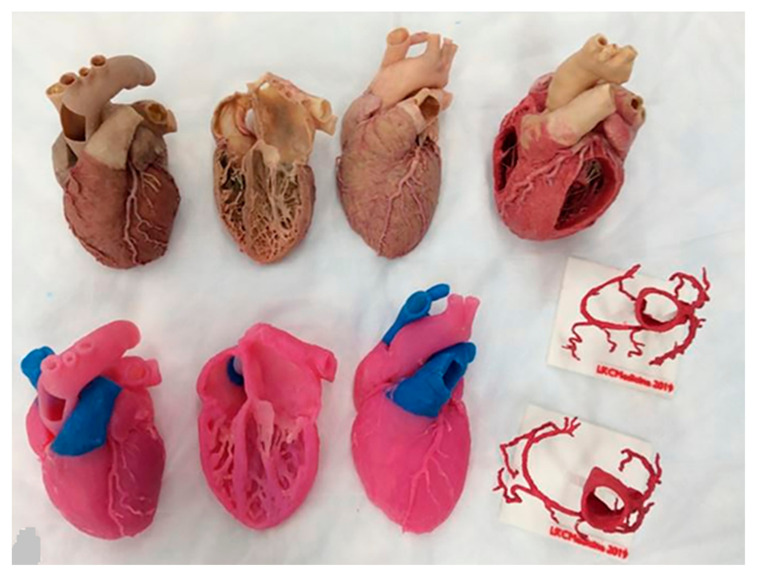
Learning materials provided to the study groups: Phase 1 materials include plastinated cardiac specimens (**top row**) and their three-dimensionally printed replicas and the coronary vessels (**bottom row**). Reprinted with permission from Mogali et al. [102].

Mogali and colleagues, in a randomized controlled trial, invited first-year medicine students to demonstrate their learning of cardiac anatomy by comparing 3D-printed cardiac models with plastinated cardiac specimens [102]. Three-dimensional printed models were generated by scanning the plastinated cardiac specimens on a 64-slice CT scanner, with the models being printed using multi-color materials (Figure 5). Overall, 32 and 31 students were randomly allocated to the plastinated cardiac and 3D-printed cardiac model groups, respectively. A significant improvement in students’ baseline knowledge was achieved by 29.7% and 31.3% of the participants in the plastinated and 3D printing groups, respectively. There was no significant difference in terms of anatomy knowledge performance between the two groups, with the mean post-test scores being 57.0 ± 13.3 and 60.8 ± 13.6 (*p* = 0.27) for the plastinated and 3D printing groups, respectively. This study shows that use of 3D-printed models did not disadvantage students in learning cardiac anatomy, thus indicating that 3D printing serves as a reliable alternative to the current teaching tools.

**Figure 6:**
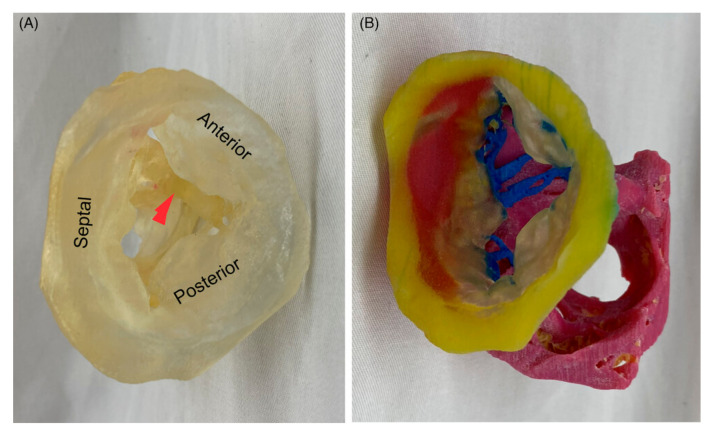
A 3D-printed model of the tricuspid valve of a human heart specimen (HH 223). (**A**) A model printed using a clear material as viewed from the atrium, with leaflets labeled and the moderator band marked with a red arrow. (**B**) A model printed using multiple colors and materials and rotated to show the subvalvular apparatus. Yellow, tricuspid annulus; transparent, mitral leaflets; blue, chordae tendinea; pink, papillary muscles. Reprinted with permission from Arango et al. [103].

**Figure 7:**
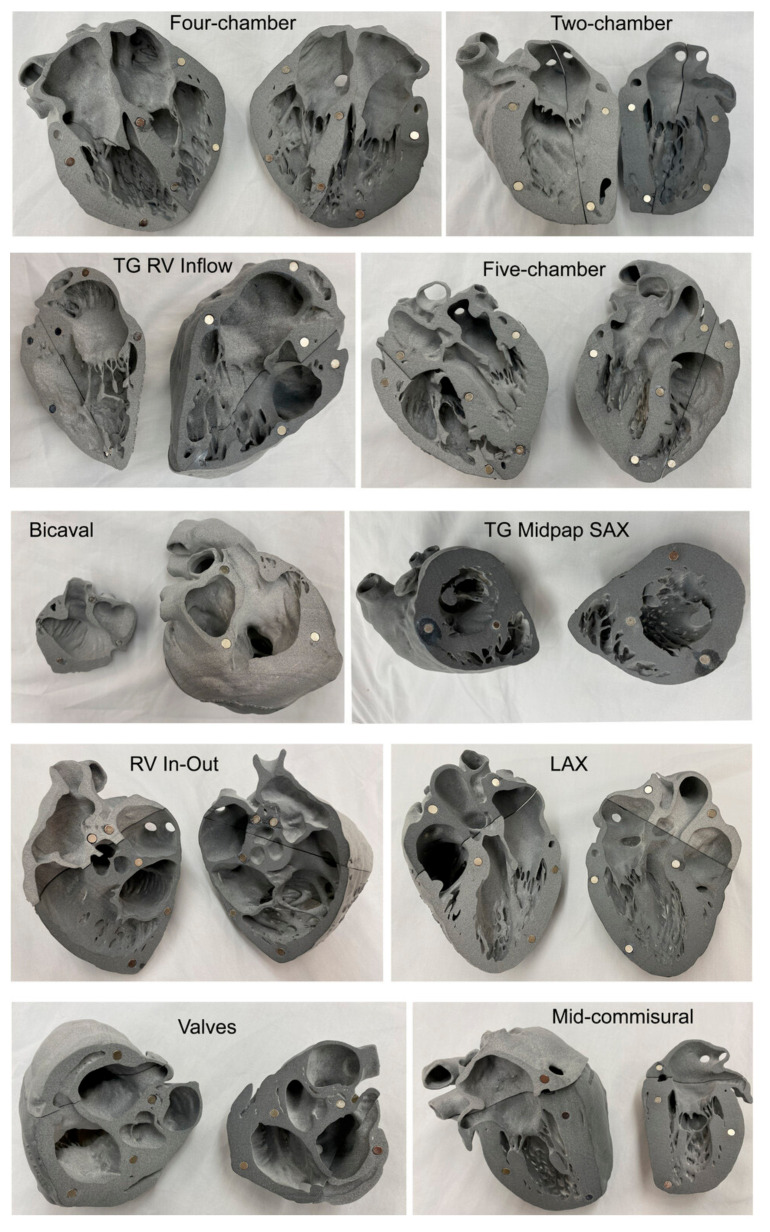
High-resolution fusion powder 3D-printed heart models representing the transesophageal echocardiography (TEE) American Society of Echocardiography (ASE)-recommended views. Each row represents two corresponding planes on each model that have been labeled accordingly. LAX, long axis; RV, right ventricle; SAX, short axis; TG, transgastric. Reprinted with permission from Arango et al. [103].

Regarding the use of 3D-printed heart and vascular models in anatomy education, the most commonly used imaging modalities are CT or MRI for the generation of 3D-printed models. In a recent study, Arango et al. reported the feasibility of creating ultra-high-resolution 3D-printed heart models using micro-CT [103]. They scanned perfusion-fixed heart specimens using 0.1 mm resolution micro-CT scanning, with the images being post-processed and segmented for 3D printing. The-3D printed heart models accurately represented cardiac walls, vessels, and the valvular and subvalvular structures of atrioventricular valves (Figure 6 and Figure 7), thus serving as useful tools to teach residents and cardiothoracic anesthesia fellows and help them to learn basic and advanced echocardiographic views, valvular pathology, and planned interventions. This study further advances the application of 3D printing technology in cardiovascular anatomy by providing ultra-high-resolution 3D models that demonstrate the finer details of cardiac anatomy.

### 4.2. Patient-Specific 3D-Printed Models: Preoperative Planning and Simulation

**Figure 8:**
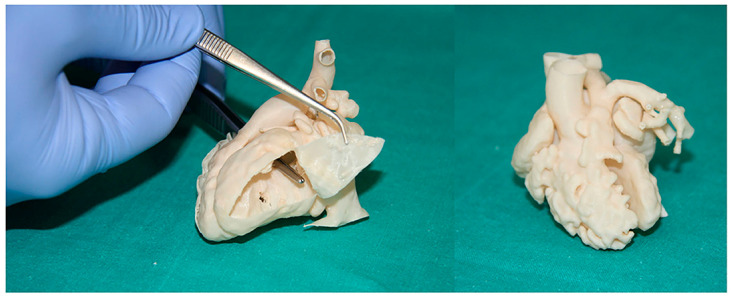
Surgical and interventional planning on 3D-printed heart models. DORV case, internal vision from the left ventricle (**left**). DORV (another case), external view (**right**). DORV-double outlet right ventricle. Reprinted with permission under the open access from Gomez-Ciriza et al. [59].

Three-dimensionally printed personalized models have been shown to play an important role in the planning and simulation of complex cardiovascular procedures, and this has been confirmed by a number of studies based on single-site investigations and multi-center reports [49,50,51,52,53,57,58,59,60,61,62,104,105,106,107,108,109,110,111,112]. Most of the current reports in this area focus on the usefulness of 3D-printed heart models in guiding CHD surgeries. This is most likely due to the complexity and wide variations of CHD, which present challenges for preoperative planning based on traditional 2D and 3D image visualizations. In contrast, 3D-printed personalized models assist cardiac surgeons in planning the treatment of CHD, with up to 50% of surgical decisions involving the aid of 3D-printed models (Figure 8) [59].

**Figure 9:**
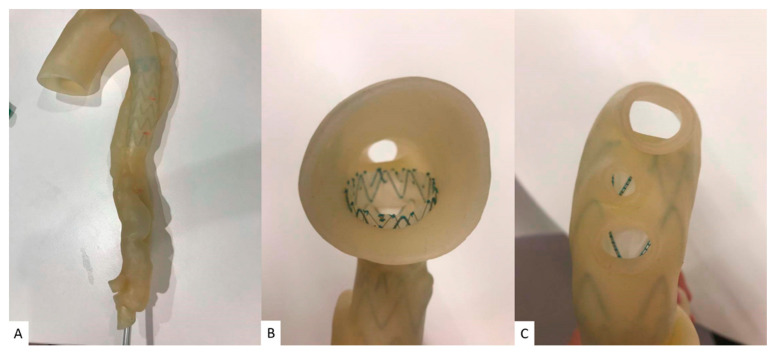
Stent graft deployed in a 3D-printed model. (**A**) Deployed stent graft visible through model wall. (**B**) Axial view from proximal arch. (**C**) Caudal view down arch vessels. Reprinted with permission under open access from Wu et al. [113].

**Figure 10:**
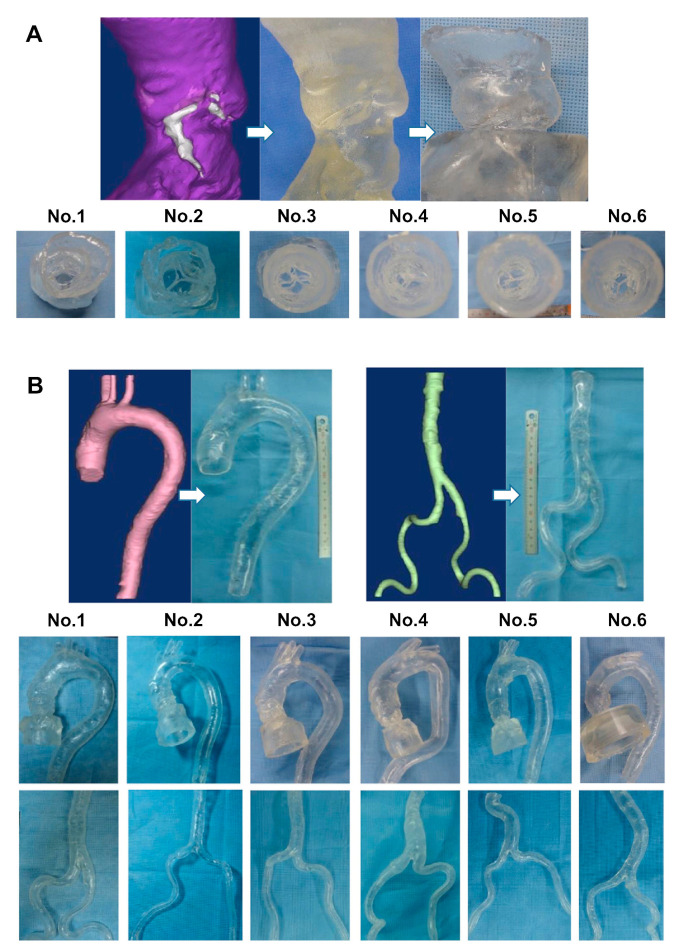

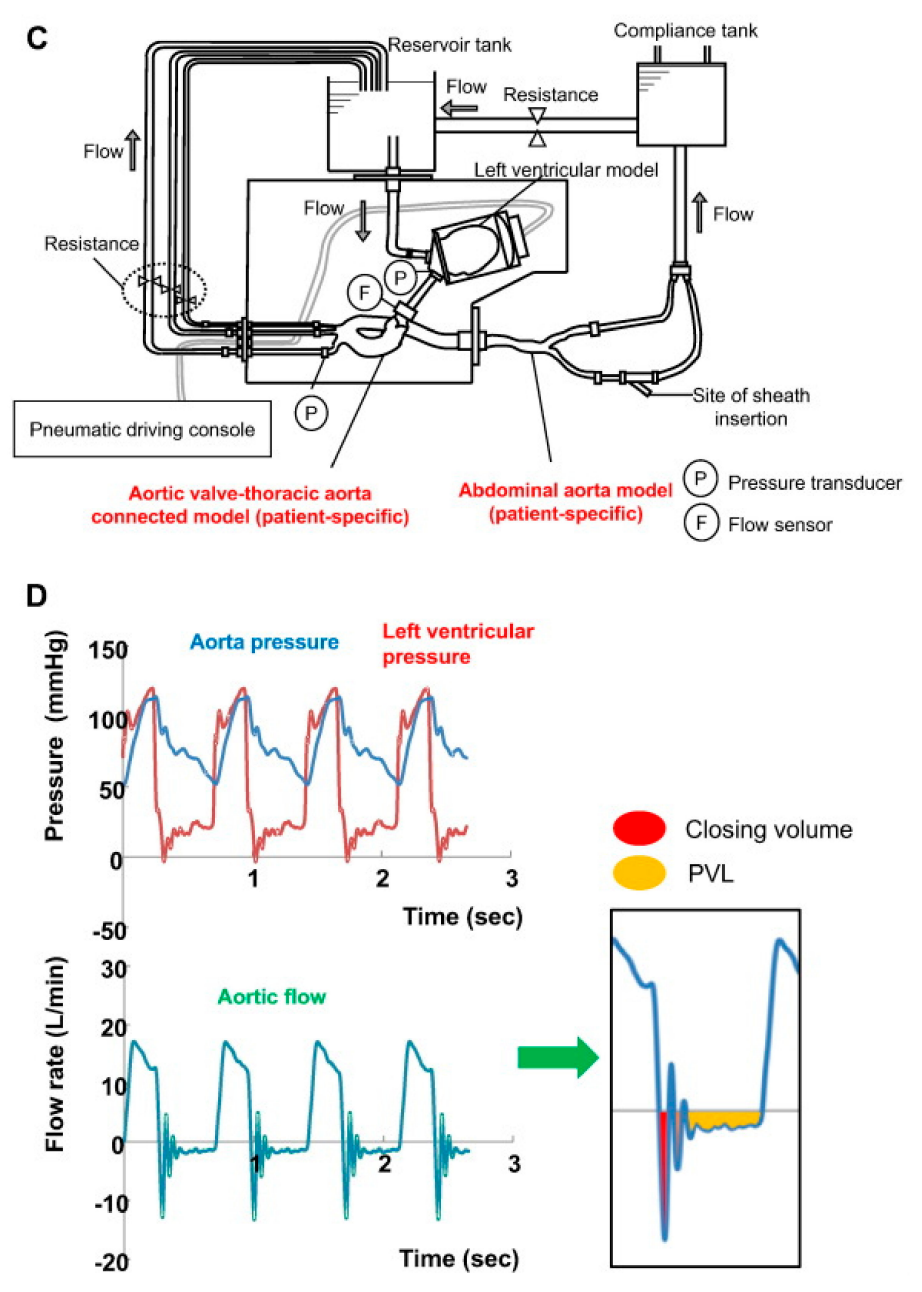
Three-dimensionally printed models with an a pulsatile circulation simulator for the assessment of transcatheter valve hemodynamics. (**A**) Aortic root with left ventricular outflow tract. (**B**) Three-dimensionally printed models of the thoracic aorta, abdominal aorta, and iliofemoral arteries. (**C**) Pulsatile circulation system. (**D**) Representative hemodynamic waveforms of left ventricular pressure (red line), aortic pressure (blue line), flow rate (green line), and the definition of closing volume and PVL (red and yellow areas). PVL = paravalvular leakage. Reprinted with permission from Tanaka et al. [115].

**Figure 11:**
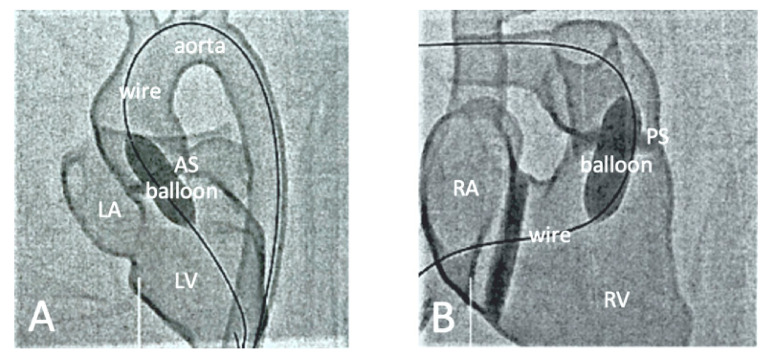
Fluoroscopic documentation of the balloon dilatation of valvular stenoses with a 3D-printed heart model. (**A**) Balloon dilatation of a valvular aortic stenosis. (**B**) Balloon dilatation of a valvular pulmonary stenosis. AS—aortic stenosis, PS—pulmonary stenosis, LA—left atrium, LV—left ventricle, RA—right atrium, RV—right ventricle. Reprinted with permission under the open access from Brunner et al. [116].

**Figure 12:**
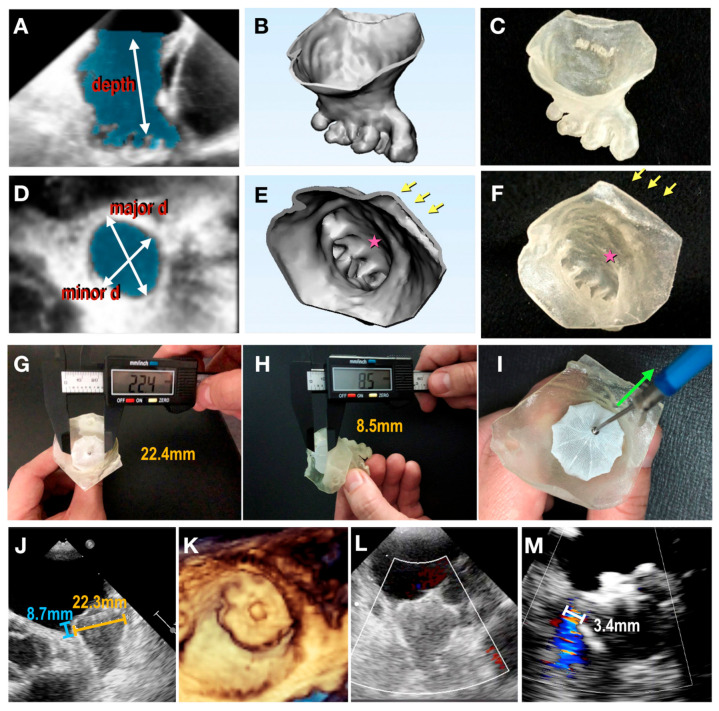
Three-dimensionally printed patient-specific models based on echocardiographic images. (**A**–**F**) From 3D transesophageal echocardiography (TEE) image to 3D physical model. (**A**,**D**) Segmentation of left atrial appendage (LAA) (shaded area) based on 3D TEE data. Measurements regarding the major and minor ostial diameters and depth of the LAA were taken. (**B**,**E**) Creation of a digital object. (**C**,**F**) Three-dimensional printed physical model made of tissue-mimicking material. Arrows denote pulmonary vein ridge; stars denote appendicular trabeculations. (**G**–**I**) Modifying the size of the 3D model. (**G**) Device compression and (**H**) protrusion in 3D model measured using a digital caliper. (**I**) Tug test for stability. (**J**) Device compression and protrusion measured in a clinical procedure. (**K**) Three-dimensional TEE en face view of final device position. (**L**) Color Doppler assessment showing no peridevice leaks. (**M**) In another case, color Doppler assessment revealed a residual leak with a jet width of 3.4 mm. Reprinted with permission under open access from Fan et al. [112].

Three-dimensionally printed models also serve as a useful tool for hands-on surgical training or the simulation of cardiovascular procedures. Three-dimensionally printed heart models can be used to simulate congenital heart surgery with high satisfaction scores. Furthermore, these models are also useful for simulating interventional cardiac procedures such as the simulation of endovascular aortic stent grafting procedures for the treatment of aortic aneurysms or aortic dissection (Figure 9) [113]; the simulation of interventional treatment for transcatheter aortic valve replacement (TAVR) for predicting the risk of coronary obstruction or complications [114,115] (Figure 10); and simulating valvular stenosis (Figure 11) [116]. Another common application of 3D-printed models is their use in guiding left atrial appendage occlude device selection, with improved outcomes and reductions in the number of complications being reported [110,112,117,118,119]. Good agreement in terms of occluder sizes was found between 3D model-based estimation and the actual device sizes, with reduced procedure time and radiation exposure to patients (Figure 12) [112]. 

**Figure 13:**
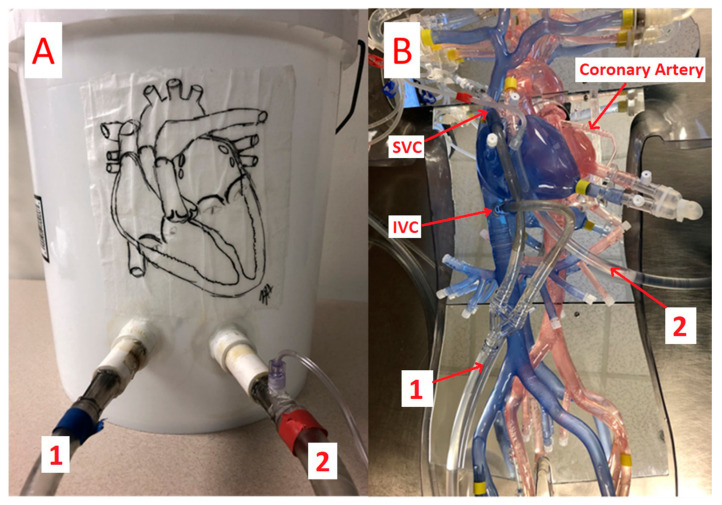
A traditional bucket patient simulator (**A**) and a 3D-printed anatomic patient simulator (**B**). 1 indicates the venous line; 2 indicates the arterial line. IVC, inferior vena cava; SVC, superior vena cava. The red arrows indicate the direction of flow. Reprinted with permission under open access from Messarra et al. [120].

Messarra and colleagues have further expanded the application of 3D printing technology to simulate coronary bypass procedures [120]. In their recent study, the researchers developed an anatomic perfusion simulator to improve students’ understanding of cannulation sites, hemodynamic flow, and anatomy related to cardiopulmonary bypass. Sixteen students were randomly allocated to two groups, with seven being allocated to the bucket group, representing the standard bucket simulator, and nine being allocated to the group representing the 3D-printed anatomic simulator with two different flow circuits (Figure 13). In addition to the simulation of cardiovascular system flow, a continuous flow pump was connected to the ports at the femoral arteries and veins to simulate the flow generated by the heart. Their results showed a significant increase in the mean test scores in the 3D printing group as opposed to the bucket group (18% vs. 3% increase). The 3D printing group achieved eight instances of true learning, while in the bucket group, there was only one. The largest gain in acuity confidence interval was found in the 3D printing group. This study shows the potential value of using 3D-printed anatomic simulators to teach perfusion students.

### 4.3. Patient-Specific 3D-Printed Models: Clinical Communication

Three-dimensional printed models have enhanced communication between colleagues, clinicians, and patients, as well as the parents of patients. This is clinically important as effective communication contributes to better patient care and clinical outcomes. Physicians usually use visual aids to provide information to patients about their disease/condition and treatment plans or options. However, it can be difficult for patients to comprehend the complexity of cardiovascular anatomy and disease, particularly in more challenging scenarios such as cases of congenital heart disease. The use of physical 3D-printed models overcomes these limitations by presenting a realistic 3D model to the patient so that their understanding of the anatomy and disease is significantly enhanced.

**Figure 14:**
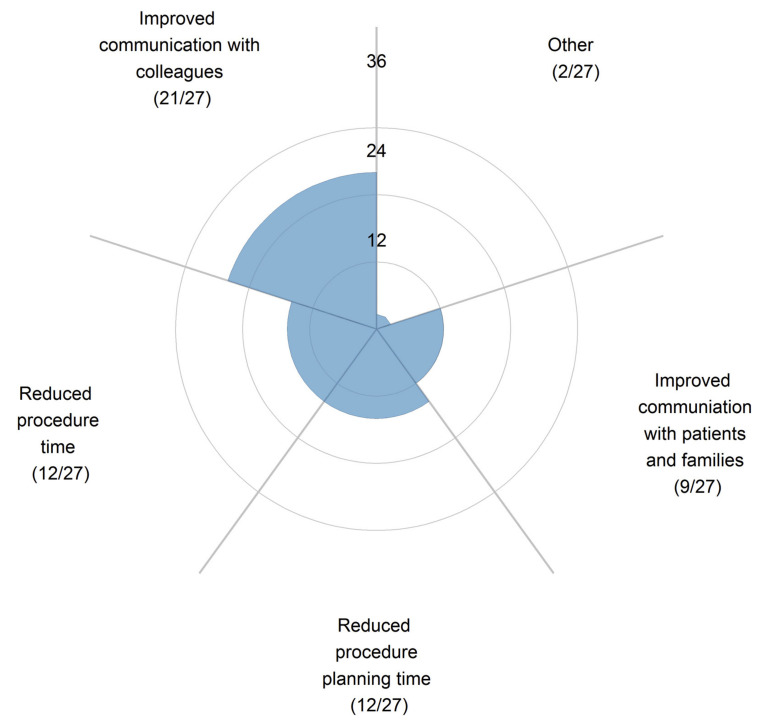
Participants’ responses on how 3D-printed cardiac models improve communication with colleagues and patients/families. Reprinted with permission under open access from Illmann et al. [122].

In their comprehensive review, Traynor et al. analyzed 19 studies about the use of 3D printing technology in patient communication [121,122,123,124,125,126,127]. These studies confirmed that 3D-printed models aided communication with patients/parents and colleagues (Figure 14) [122].

**Figure 15:**
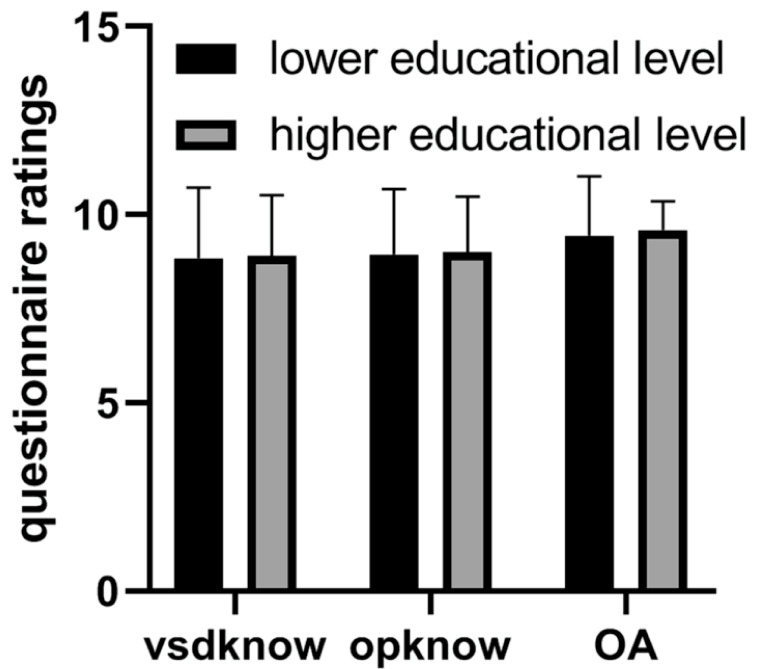
Comparison of questionnaire results regarding different education levels (which did not show significant differences). vsdknow: VSD knowledge, opknow: operation knowledge, OA: overall understanding. Reprinted with permission under open access from Deng et al. [128].

**Figure 16:**
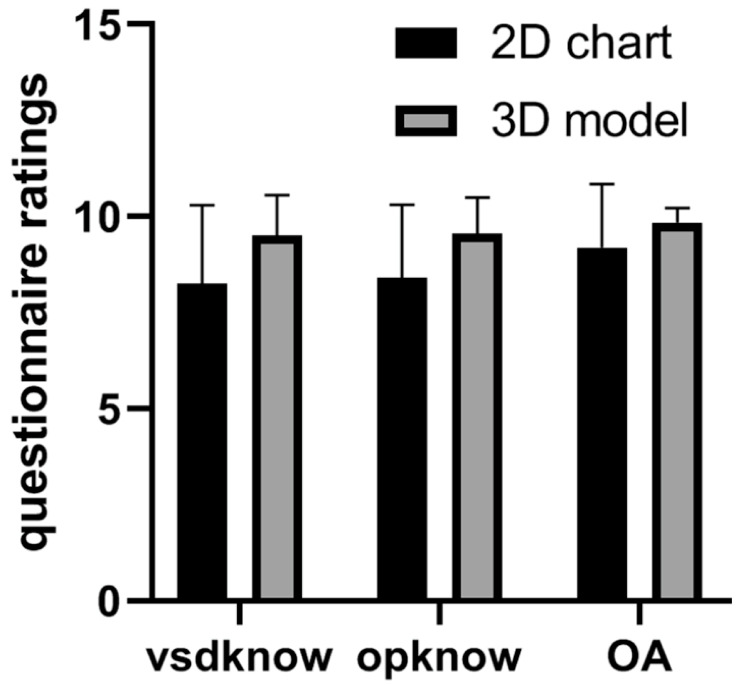
Comparison of questionnaire responses between the two groups in the aforementioned study conducted by Deng et al. Guardians’ understanding of both VSD and operation knowledge was found to be significantly higher in the 3D printing group, although no significant differences were found in the overall ratings. vsdknow: VSD knowledge, opknow: Operation knowledge, OA: overall understanding. Reprinted with permission under open access from Deng et al. [128].

Deng et al., in a randomized controlled trial, studied the clinical value of using 3D-printed heart models in surgical contexts related to congenital heart surgery [128]. The guardians of 40 patients who were treated via ventricular septal defect repair were invited to participate in the study, with 20 guardians being allocated to two groups: the study group and a control group. The study group received the same information as the control group (such as ventricular septal defect condition and surgical implications) as well as 3D-printed models to help with the explanation of these details. This study’s results showed significant improvements in participants’ understanding of congenital heart disease and surgical procedures in the study group when compared to the control group (Figure 15 and Figure 16).

### 4.4. Patient-Specific 3D-Printed Models: Optimizing CT Protocols

**Figure 17:**
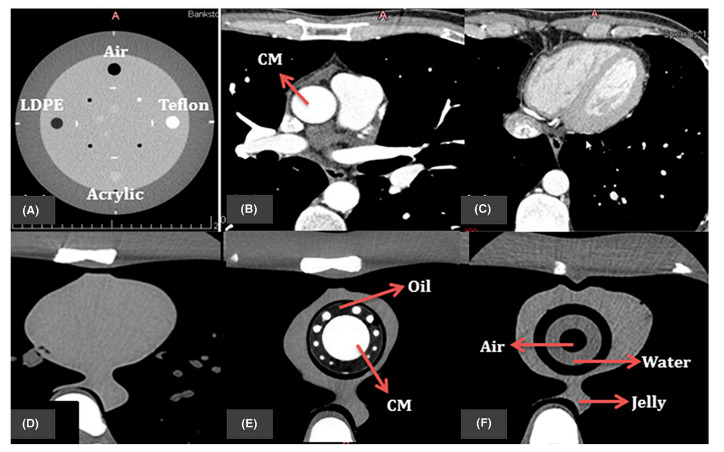
The resulting axial CT of (**A**) four inserts in Catphan@ 500 phantom; (**B**,**C**) patient image datasets for cardiac CT; (**D**) original cardiac insert of anthropomorphic chest phantom; (**E**,**F**) 3D-printed cardiac insert phantom with the contrast materials (CM), oil, air, water, and jelly segmented all labeled. Reprinted with permission under the open access from Abdullah et al. [139].

It is well known that CT is associated with relatively high radiation doses; thus, minimizing radiation exposure is clinically significant, given the widespread use of CT in daily practice. The traditional reliance on the use of commercial phantoms to study optimal CT protocols has become less practicable due to expensive costs and the lack of simulations regarding the situations of individual patients. The use of 3D-printed models to optimize cardiovascular CT scanning protocols by representing realistic anatomical structures of cardiovascular systems or organs has become a new research direction [129,130,131,132,133,134,135,136,137]. The current literature shows the feasibility of using personalized 3D-printed heart or vascular models to study CT protocols with minimized radiation doses. Despite only a few studies on this topic being available in the literature, the use of patient-specific 3D-printed heart and vascular models show promising results for the optimization of CT protocols [132,134,135,136,137,138,139,140]. Abdullah et al. and Morup et al. reported the feasibility of simulating human heart and vascular tissue properties using different materials, concluding that these simulations could potentially be used to optimize cardiac CT protocols (Figure 17) [139,140]. 

**Figure 18:**
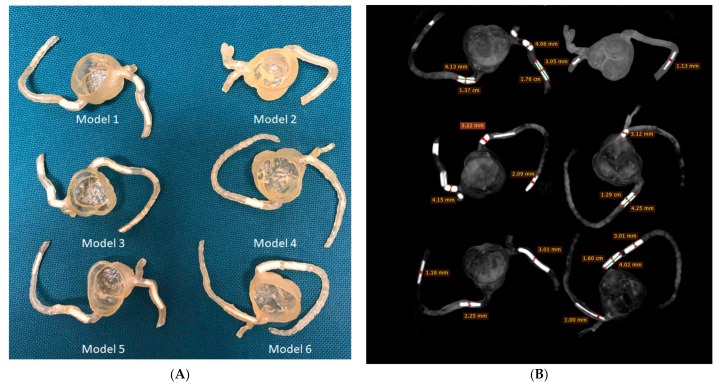
Three-dimensional printed patient-specific coronary models based on the simulation of calcified plaques in the coronary arteries. (**A**) Three-dimensional printed models (*n* = 6) with simulated calcified plaques in coronary artery branches. (**B**) Measurements of plaque dimensions on 2D maximum-intensity projection images using 0.5 mm slice thickness. Reprinted with permission under open access from Sun et al. [130].

Sun et al. developed 3D-printed aorta and coronary artery models to study the optimal CT protocols in imaging aortic dissection [135,137] and coronary plaques and stents [130,131,132,138]. Patient-specific coronary artery models were developed to represent realistic coronary artery trees via the simulation of calcified plaques in different locations of coronary arteries showing extensive calcification (Figure 18) [130]. This allowed for the study of different CT scanning protocols and the visualization of calcified plaques, which always present challenges in accurately assessing the degree of coronary stenosis due to the blooming artefacts associated with heavy calcification.

**Figure 19:**
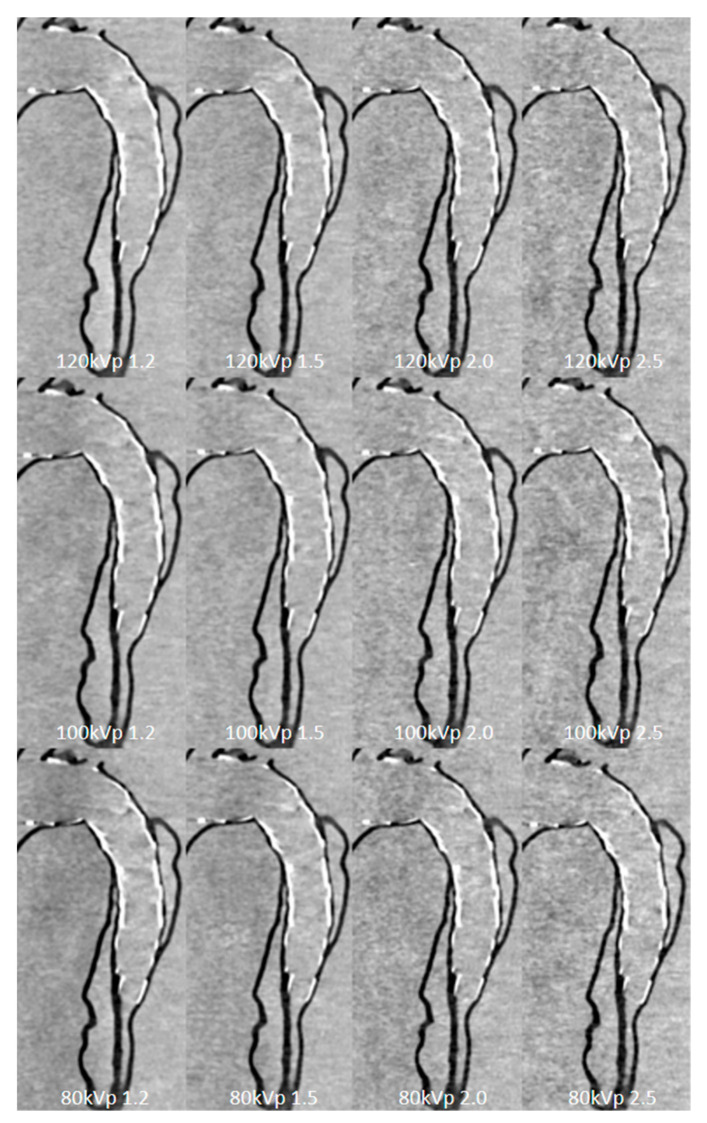
Sagittal reformatted images of CTA protocols. When kVp was decreased to 80, image noise increased with the use of high-pitch protocol values of 2.0 and 2.5. CTA: computed tomography angiography; kVp: kilovoltage peak. Reprinted with permission under open access from Wu et al. [113].

Three-dimensional printed aorta models can be used to optimize aortic CT angiographic protocols for patients with type B aortic dissection after being treated via endovascular stent graft repair (EVAR) [113,135]. CT angiography is routinely used to follow up patients following EVAR treatment for aortic dissection; thus, reducing radiation doses is necessary due to each patient receiving repeated CT scans. Three-dimensional printed aortic dissection modeling based on the simulation of endovascular repair has been shown to optimize CT protocols and reduce radiation doses by more than 20% while preserving diagnostic image quality (Figure 19) [113]. These early results have laid a good foundation for further experiments on the use of 3D-printed models for studying CT protocols.

### 4.5. The Use of 3D-Printed Devices in Treating Cardiovascular Disease

**Figure 20:**
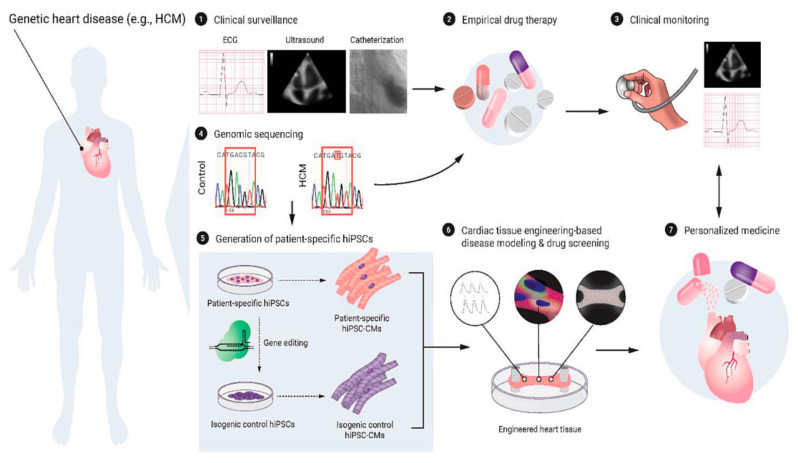
Diagram highlighting 3D bioprinting applications. Reprinted with permission under open access from Häneke et al. [158]. CM—cardiomyocyte; HCM—hypertrophic cardiomyopathy; hiPSC—human inducible pluripotent stem cell.

Patient-specific 3D-printed devices have been used in treating cardiovascular disease, and promising clinical outcomes have been achieved. It has been reported that 3D-printed devices including stents and valves have improved vessel patency and quality of life in cases of pulmonary stenosis, coronary stenotic lesions, and complex valve pathologies [104,107,110,112,117,118,119,141,142]. The factors that affect the long-term safety of using 3D-printed devices to model stents in patients with coronary artery disease, which include biocompatibility, thrombosis, degradation and mechanical stability, long-term durability and performance, vessel injury, adverse events and complications, patient suitability, and anatomical variability, should be considered [54,143]. Three-dimensional bioprinting is a promising technology advancing the applications of 3D-printed models to another level, although most of the current applications of 3D bioprinting in cardiovascular disease are still in their early stages of development [144]. Significant progress has been achieved over the last decade regarding the use of 3D-printed heart valves in treating valvular heart disease and 3D-printed cardiac patch and heart models in treating myocardial infarction, and heart failure [145,146,147,148,149,150,151,152,153,154,155,156,157,158,159]. Figure 20 presents an overview of the use of 3D bioprinting in cardiovascular disease [159]. Another emerging area is 4D printing in cardiovascular disease, which either utilizes smart materials to print models that are deformed or reshaped in response to stimuli or printing 3D micro-tissues to form the expected functional tissue structures and maturing them over time [159]. Please see the several excellent review articles on the applications of 3D bioprinting in cardiovascular disease [148,150,152,158,159,160].

**Figure 21:**
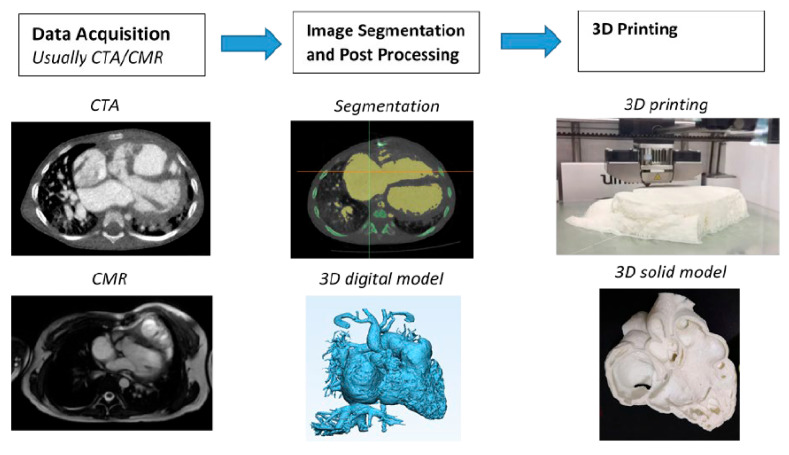
Steps involved in creating 3D-printed models using cardiac CT and MR data. CTA—computed tomography angiography; CMR—cardiac magnetic resonance. Reprinted with permission under open access from Sun et al. [52].

Of the imaging modalities used for developing personalized 3D-printed heart and vascular models, cardiac CT and MRI are the most commonly used imaging modalities in 3D printing applications related to cardiovascular disease. Figure 21 is a flow chart showing the steps that need to be followed to generate 3D printed models. Of the various image processing software tools, Mimics (19.0-25.0) (Materialise, Leuven, Belgium) is the most common commercially available software for image post-processing and segmentation, while the open-source tool 3D Slicer (5.6.1) represents another commonly used software in 3D printing [161,162].

**Figure 22:**
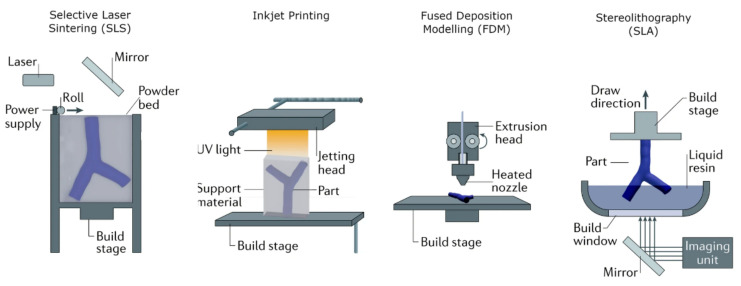
Commonly used 3D printers. Reprinted with permission under open access from Gharleghi et al. [162].

**Figure 23:**
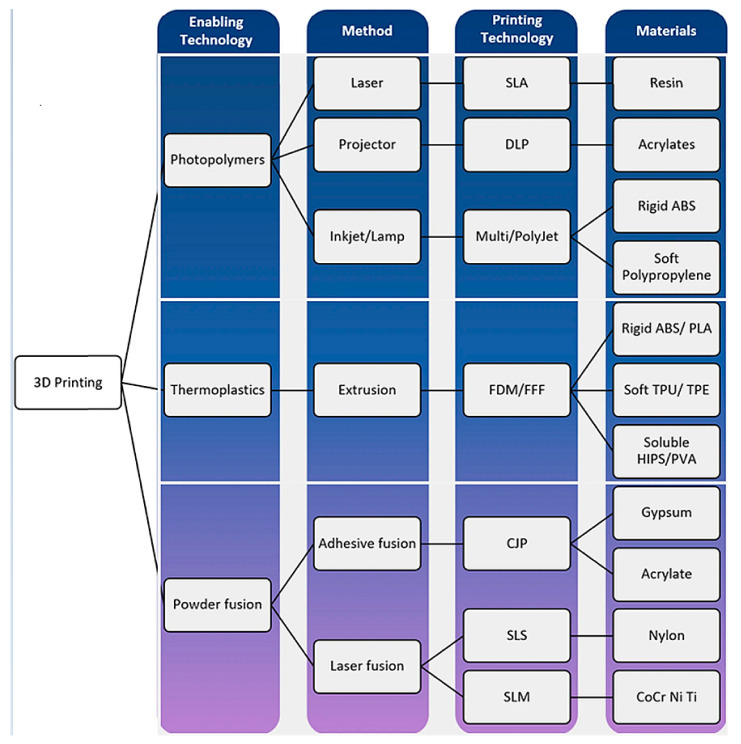
Three-dimensional printing technologies and materials. FDM—fused deposition modeling, SLA—stereolithography, DLP—digital light processing, ABS—acrylonitrile–butadiene–styrene, PLA—polylactic acid, TPU—thermoplastic polyurethane, TPE—thermoplastic elastomers, HIPS—high-impact polystyrene, PVA—polyvinyl alcohol, CJP—color jet printing, SLS—selective laser sintering, SLM—selective laser melting, CoCr—cobalt–chromium, Ni—nickel, Ti—titanium. Reprinted with permission under open access from Gharleghi et al. [162].

Figure 22 shows 3D printers that are commonly used in cardiovascular practice, while Figure 23 provides an overview of the 3D printing technologies and materials that can be used to generate patient-specific cardiovascular models [162]. The costs of printing cardiovascular models depend on the size of the model and the materials used (soft versus rigid versus multi-color materials), with costs being variable, ranging from less than USD 100 to more than USD 1000 [161,162].

### 4.6. Cardiac CT: CT-Derived FFR

The main limitation of using coronary CTA in predicting the functional significance of coronary stenosis has been addressed using CT-derived fractional flow reserve (FFRCT), and promising clinical outcomes derived from the use of FFRCT have been achieved in many single-site and multi-center studies.

FFR is the reference method for determining lesion-specific ischemia. The clinical value of FFR-guided patient management strategies has been well studied, with beneficial effects being reported in many studies [66,163,164,165,166,167,168,169,170]. Maznyczka et al., in their recent meta-analysis, analyzed eight randomized controlled trials to compare outcomes between FFR-guided versus ICA-guided management strategies for patients with obstructive CAD [171]. Their analysis did not show significant differences in all-cause mortality (3.5% vs. 3.7%, *p* = 0.98), myocardial infarction (5.3% vs. 5.9%, *p* = 0.69), and unplanned revascularization (7.4% vs. 7.9%, *p* = 0.37). However, in the FFR-guided group, the number of patients undergoing planned revascularization either by stent or surgery was found to be significantly lower than that in the invasive coronary angiography (ICA)-guided group (mean difference: 14, 95% CI: 3–25%, *p* < 0.001). They concluded that an FFR-guided approach could reduce revascularization procedures by up to 25%, hence providing significant clinical benefits to patients and also in terms of the efficient use of local health resources.

However, FFR is not widely used in clinical practice due to its invasive nature and the potential risks associated with coronary interventional procedures. The use of FFRCT has undergone rapid developments over the last few decades, from early studies to more recent ones, as evidenced by the incorporation of deep learning models into simulations of fractional flow reserve based on coronary CTA images [33,34,35,36,37,38,39,40,41,42,43,44,45,46,47,48]. 

**Figure 24:**
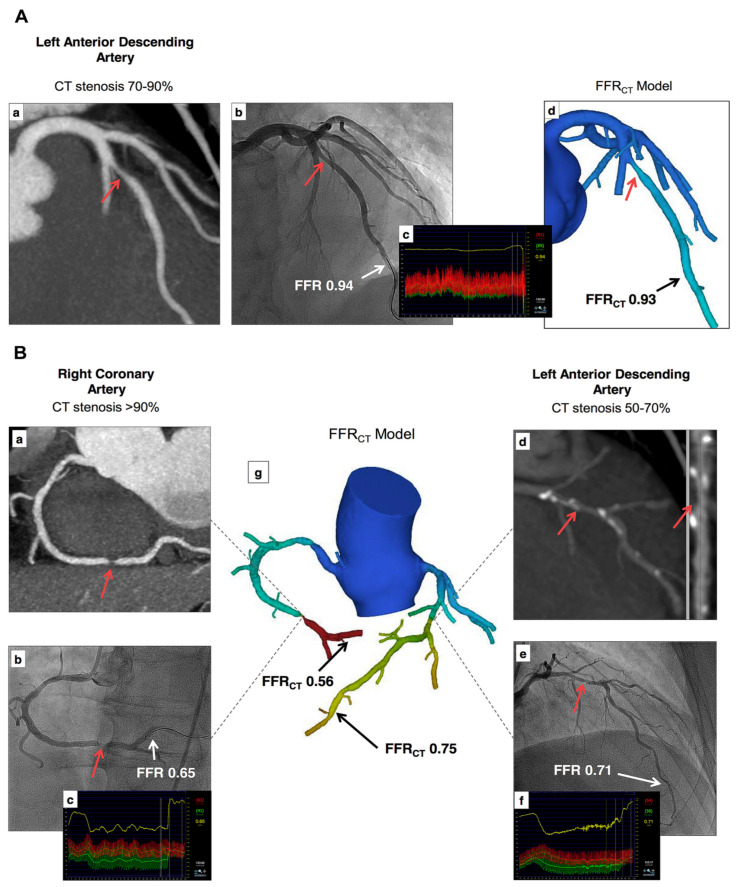
Examples of FFRCT in assessing the hemodynamic significance of coronary lesions at three main coronary arteries (**A**,**B**). Coronary CT angiography shows significant stenoses on the left anterior descending artery (LAD), right coronary artery (RCA), and left circumflex (LCx), while FFRCT shows ischemia at RCA and LCx but not at LAD, as the FFRCT value is more than 0.80. This was confirmed by invasive FFR measurements, as shown in (**A**(**c**)) and (**B**(**c**,**f**)). (**a**,**b**) in image (**A**), (**a**,**b**,**d**,**e**) in image (**B**) refer to stenotic lesions of RCA and LAD on coronary CT angiography and invasive FFR measurements, respectively, while ((**A**)**d**,(**B**)**g**) indicate FFRCT measurements at these coronary arteries. Reprinted with permission from Norgaard et al. [43].

FFRCT has unique advantages over standard coronary CTA by visualizing the coronary stenosis and assessing its hemodynamic significance, thus allowing for a combined anatomic–physiologic assessment of CAD. Representative multi-center studies including DISCOVER-FLOW, NXT, DeFACTO, and NOVEL-FLOW published more than 10 years ago confirmed that FFRCT has an enhanced diagnostic value, as well as especially enhanced specificity in detecting hemodynamically significant CAD when compared to standard coronary CTA based on lumen assessment (Figure 24) [41,42,43]. Recent randomized controlled trials have further validated these findings. The PLATFORM study and FORECAST trials involved multiple sites, with the results showing that use of FFRCT can guide patient management and resource use [44,45,46,47,48,49]. The results from the FORECAST trial did not show significant differences in terms of major adverse cardiac and cerebrovascular events between the study and standard groups, but a significant reduction in the use of invasive coronary angiography (19% vs. 25%, *p* < 0.01) was observed in the study group after selective FFRCT use [48]. The ADVANCE registry also reported that FFRCT modified the treatment of patients with stable chest pain in two thirds of cases when compared to coronary CTA alone, and it was associated with significantly lower negative rates of ICA [46].

The TARGET trial was a recently conducted randomized controlled trial involving 1216 patients from six Chinese medical centers [172]. This trial mainly focused on the on-site FFRCT-guided management of patients using machine learning (*n* = 608) with stable CAD compared to standard care (*n* = 608 patients). Their results showed that FFRCT reduced the proportion of patients undergoing ICA procedures without showing any improvements in quality of life, but increased revascularization rates compared to the standard care group were found (49.7% vs. 42.8%, *p* = 0.02). There was no significant difference in major adverse cardiovascular events at 1 year follow-up between the two groups. This study also reported cost savings with use of FFRCT, mainly because of a reduction in interventional procedures. However, due to the differences between the reimbursement models of China and other countries, further investigations into the cost-effectiveness of FFRCT in other healthcare systems is needed.

### 4.7. Cardiovascular CT: VR, AR, and MR

Rapid developments in 3D innovative technologies, including VR, AR, and MR, have further advanced the roles of traditional image visualizations in cardiovascular disease and patient care, with increasing studies proving their educational and clinical value in cardiovascular medicine. Due to complex cardiovascular anatomical and pathological aspects and the importance of lifelong learning and training required to deliver high-quality standards in cardiovascular care, these 3D visualizations serve as useful tools for healthcare providers and patients [64,65,66,67,68,105,173,174,175,176,177,178,179,180,181,182].

**Figure 25:**
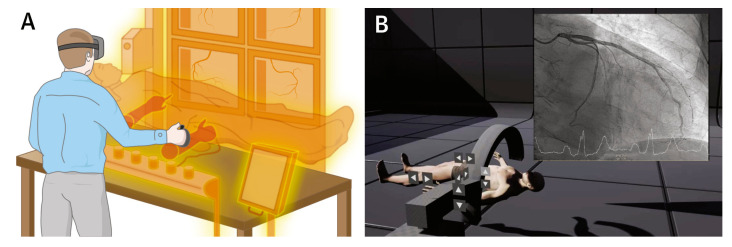
VR completely immersing the user in a virtual 3D space. (**A**) User is completely immersed in a virtual 3D space with use of a head-mounted display. (**B**) A real-life example of VR application allowing trainees to perform virtual coronary angiograms. Reprinted with permission from Jun et al. [173].

**Figure 26:**
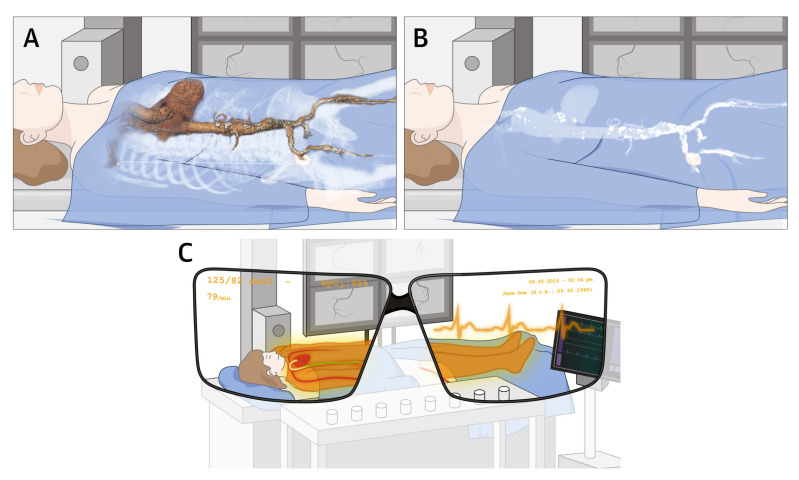
AR integrates superimposed virtual elements into a real-world environment. (**A**) 3D CT image of a patient’s vasculature could be imaged by an operator or (**B**) vascular calcifications could be focused to guide the best puncture site and avoid complications during the procedure. (**C**) AR superimposes virtual elements into a real-world environment. Reprinted with permission from Jun et al. [173].

VR allows the user to completely immerse themselves in a 3D virtual environment, usually using a head-mounted display (Figure 25). In contrast, AR integrates virtual objects into a real-world environment, thus enabling the user to interact with virtual models, improving the simulation and management of complex cardiovascular procedures. MR represents an advancement of AR, and it is a recently developed technology that mainly involves overlaying virtual objects on real world settings (Figure 26) [172]. Extended reality (XR) is a new term used to encompass all three of these tools (VR, AR, and MR). The use of VR and AR and MR in medical education and training has been confirmed to improve the understanding of 3D relationships in all medical disciplines [173,174,175,176,177,178,179,180].

Barteit et al., in a systematic review, analyzed 27 studies, comprising a total of 956 participants, about the usefulness of VR, AR, and MR in medical education [67]. The participants included all types of healthcare professionals, of which medical students (59.9%) and residents (30.2%) represented 90% of them. AR and MR were mainly implemented in surgery training (48%) and anatomy learning (15%). Users showed great enthusiasm and enjoyment in learning anatomy with the use of VR-, AR-, and MR-based head-mounted devices. This review and other studies highlight the effectiveness of using innovative 3D tools to enhance the learning of cardiac anatomy and pathology, with non-inferior results when compared to conventional teaching methods being found [66,67,68,175].

In the context of cardiovascular disease, the applications of VR and AR mainly lie in the field of interventional cardiology for the purposes of procedural simulation and training, such as planning or simulating TAVR, CHD, or valvular interventions; left atrial appendage occlusion; and/or cardiac ablation procedures [105,174,175,176,177,178,179,180,181,182]. Studies have shown that the use of VR-/AR-guided approaches can result in more successful attempts with fewer distance errors, shorter navigation times, and shorter path lengths during valvular interventional procedures [65,105]. These techniques are showing great potential for integration into cardiac catheterization laboratories, although more robust studies including more patients are needed.

VR, AR, and, more recently, MR are being successfully used in CHD contexts, covering different aspects, from education to the training and simulation of interventional procedures [176,181,182,183]. Due to the variety of abnormalities associated with CHD presenting challenges to understanding its complex cardiac anatomy and pathology, VR and AR tools demonstrate superior advantages over traditional 2D/3D visualizations in medical education, as well as surgical or interventional planning regarding CHD [182,183,184]. VR was found to be the preferred display system in visualizing CHD, as assessed by students and healthcare professionals, in [184,185]. Lau et al. compared VR with 3D-printed heart models in four different CHD cases through conducting a survey-based study involving 29 participants [182]. Both the VR and 3D-printed models were comparable in terms of their degrees of realism; however, VR was ranked as more useful than 3D-printed models in education and preoperative planning. The same research group extended their study to compare MR with 3D-printed models in two selected CHD cases, as assessed by 34 cardiac specialists and physicians [183]. MR was ranked as the best modality, rather than 3D-printed models and original DICOM images, in most clinical applications, including the demonstration of complex CHD lesions, enhancing depth perception, the learning of cardiac pathology, and facilitating preoperative planning (*p* < 0.05). In contrast, 3D-printed models were ranked as the best tool for facilitating communication with patients.

**Figure 27:**
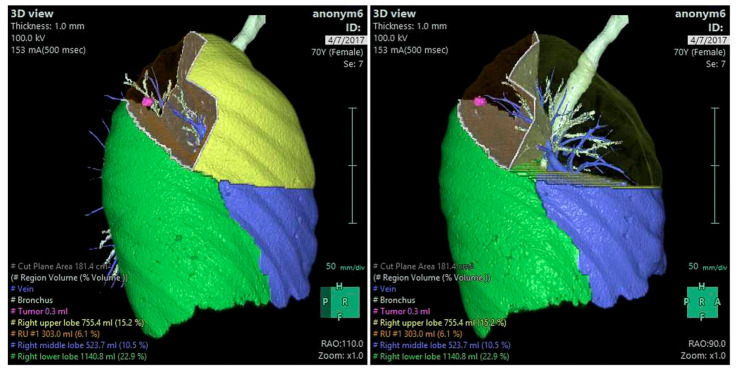
Virtual simulation of segmentectomy. Reprinted with permission under open access from Rd et al. [186].

Rad and colleagues reviewed the applications of XR in thoracic surgery by conducting a systematic review of VR and AR in three main applications related to thoracic surgery: virtual simulations for training, preoperative planning, and intraoperative guidance [186]. They identified 21 studies published from 2007 to 2019, leading to the consideration of 1570 patients. A total of 7, 11, and 9 studies were analyzed in relation to their focus on training, preoperative planning, and intraoperative assistance, respectively. This review represents the first updated analysis of XR (VR and AR) in thoracic surgery and shows the potential of using these technologies in this field. XR increases procedural accuracy and surgical confidence through improving our intraoperative understanding of patient anatomy and the simulation of complex surgical procedures (Figure 27). An analysis of these studies also confirmed that the use of VR/AR is more accurate than conventional methods in establishing reference markers for surgical navigation [186].

## 5. Cardiovascular CT: AI/ML/DL

The role of cardiovascular CT imaging in clinical practice continues to grow following rapid technological advancements, and the introduction of AI, ML, and DL algorithms into cardiovascular practice will further enhance its clinical value, from optimizing day-to-day workflows to supporting clinical decision making [69,70,71,72,73]. AI has been applied in all aspects of cardiovascular CT imaging, from the optimization of data acquisition, image post-processing, and segmentation to improving image quality, automated detection, and the quantification of cardiovascular lesions such as plaques and stenosis. AI could also help to reduce inappropriate imaging studies, thus assisting clinicians to adhere to practice guidelines and ever-changing appropriate use criteria [69].

### 5.1. AI/ML/DL in Coronary Calcium Scoring

Coronary calcium scoring is a routine procedure performed in clinical practice to provide risk stratification for coronary artery disease; however, the quantification of calcium scores could be a time-consuming job, as it still requires the involvement of human observers in interpreting the non-contrast cardiac CT images. AI, specifically DL tools, has been increasingly used for the automated quantification of calcium scores, showing high accuracy compared to manual interpretation [40,187,188,189,190]. Wang et al. tested their DL algorithm in 140 patients suspected of CAD, and no significant differences were found in terms of the Agatston, mass, and volume scores between the DL and manual method [187]. There was excellent agreement between the two methods regarding the Agastston score categories and cardiac risk stratification, although 16% of patients were reclassified. Their findings were confirmed by a more recent study that included more cardiac CT data acquired from different scanners [190]. Mu et al. applied a DL model to spectral coronary CT angiography and non-contrast CT data from 365 patients, which were used in the training and validation datasets, while data pertaining to 240 cases were used for the independent testing of the DL model [191]. CT images were acquired from Philips (IQon Spectral CT and iCT) and GE (Revolution) scanners using different protocols. There was an excellent positive correlation in Agatston score and also excellent agreement in risk categorization between the DL model and human observer. Another advantage of their study over previous ones is the fact that it validated the DL model on different CT data from spectral and single-energy scanners, and the results showed no significant difference regarding the scanner type, sex, and section thickness (*p* > 0.05).

### 5.2. AI/ML/DL in Coronary Artery Disease

**Figure 28:**
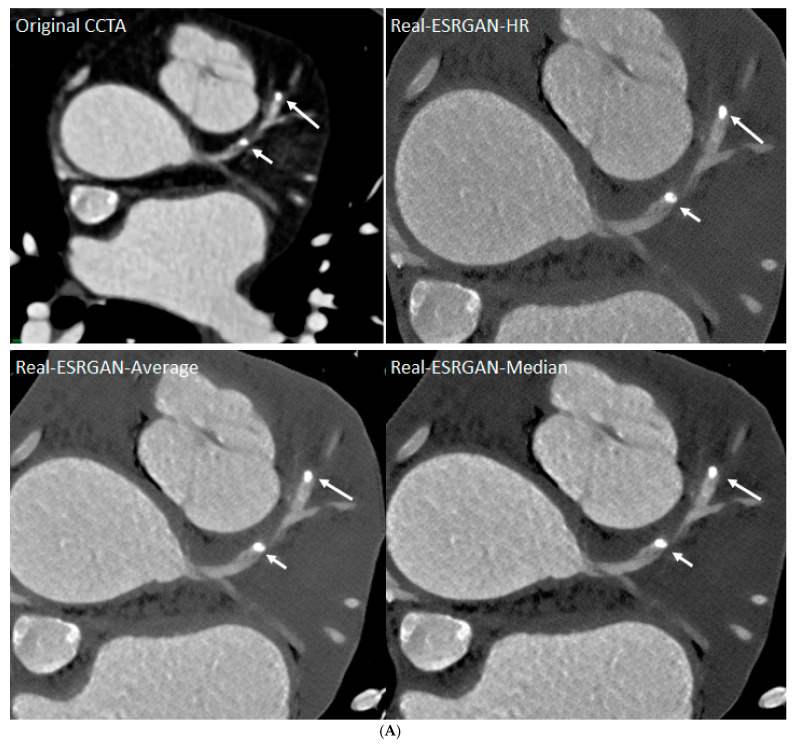

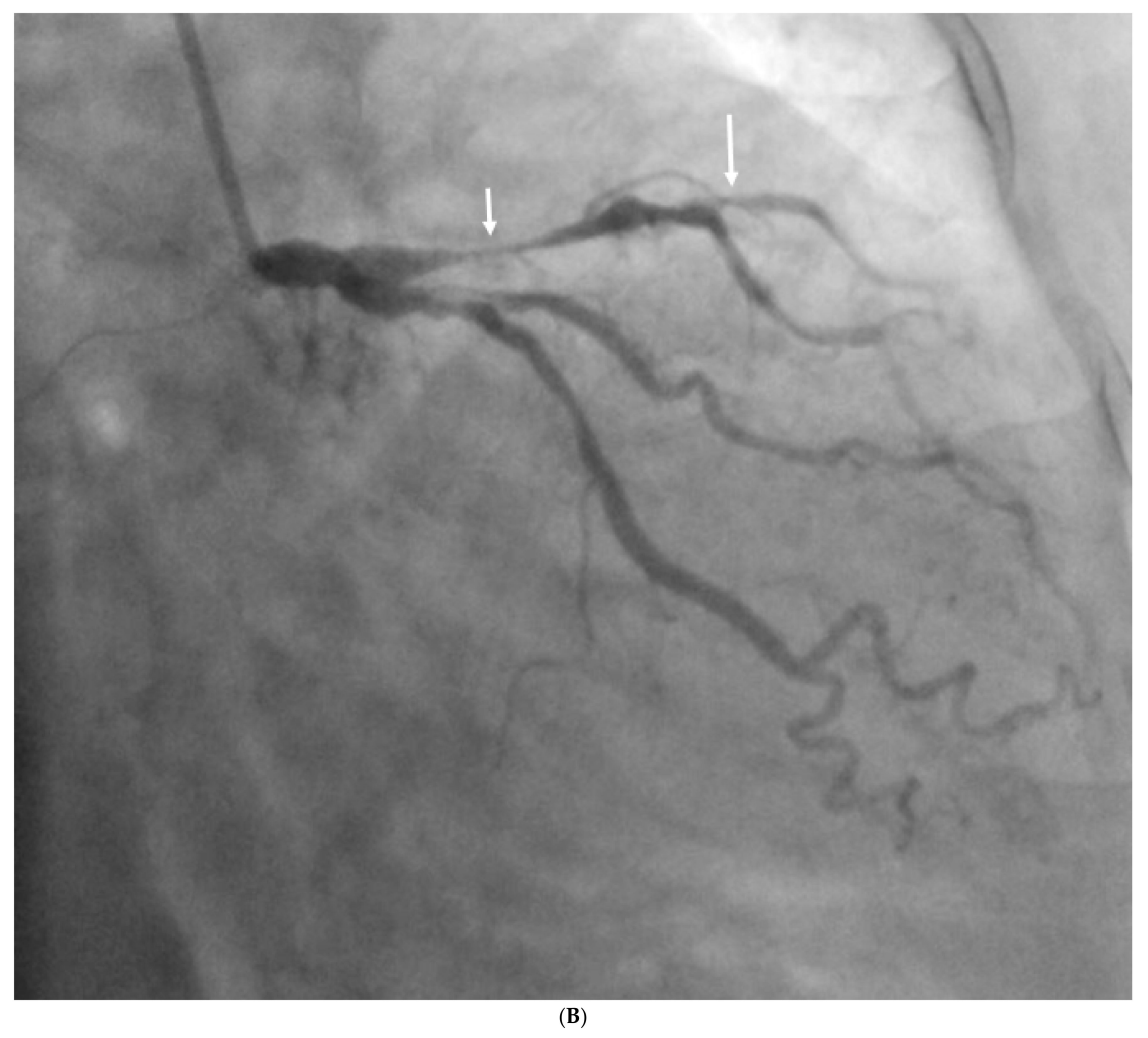
Multiple calcified plaques at the left anterior descending artery (LAD) in a 72-year-old female. Coronary stenoses were measured at 80%, 78%, 72%, and 70% corresponding to the original CCTA, Real-ESRGAN-HR, Real-ESRGAN-Average and Real-ESRGAN-Median images (short arrows in (**A**)), respectively. ICA (short arrow in (**B**)) confirms 75% stenosis. The distal stenoses at LAD due to calcified plaques were measured at 70%, 50%, and 51% stenosis on original CCTA, Real-ESRGAN-HR, and Real-ESRGAN-Average images but measured at 45% on Real-ESRGAN-Median images (long arrows in (**A**)). ICA confirmed the only 37% stenosis (long arrow in (**B**)). CCTA—coronary computed tomography angiography; ESRGAN—enhanced super-resolution generative adversarial network; HR—high resolution; ICA—invasive coronary angiography, Real-ESRGAN—real-enhanced super-resolution generative adversarial network. Reprinted with permission under open access from Sun and Ng [89].

The use of advanced AI algorithms has shown significant improvements in assessing calcified plaques, with the results of some studies showing reductions in the number of false-positive rates [88,89,192,193,194]. In our recent study, we applied a fine-tuned DL model, the real-enhanced super-resolution generative adversarial network (Real-ESRGAN), to process data pertaining to 50 coronary CTA cases from patients with a total of 184 calcified plaques [88,89]. Measurements of coronary lumen stenosis from AI-processed images were compared to those from original coronary CTA, with ICA being used as the reference method. The Real-ESRGAN -processed images showed improvements in terms of specificity and positive predictive value at all the three main coronary arteries, along with significant reductions in false-positive rates (Figure 28). This DL model has significant clinical value, as reducing false-positive rates will contribute to avoiding unnecessary downstream testing, mainly in ICA procedures.

**Figure 29:**
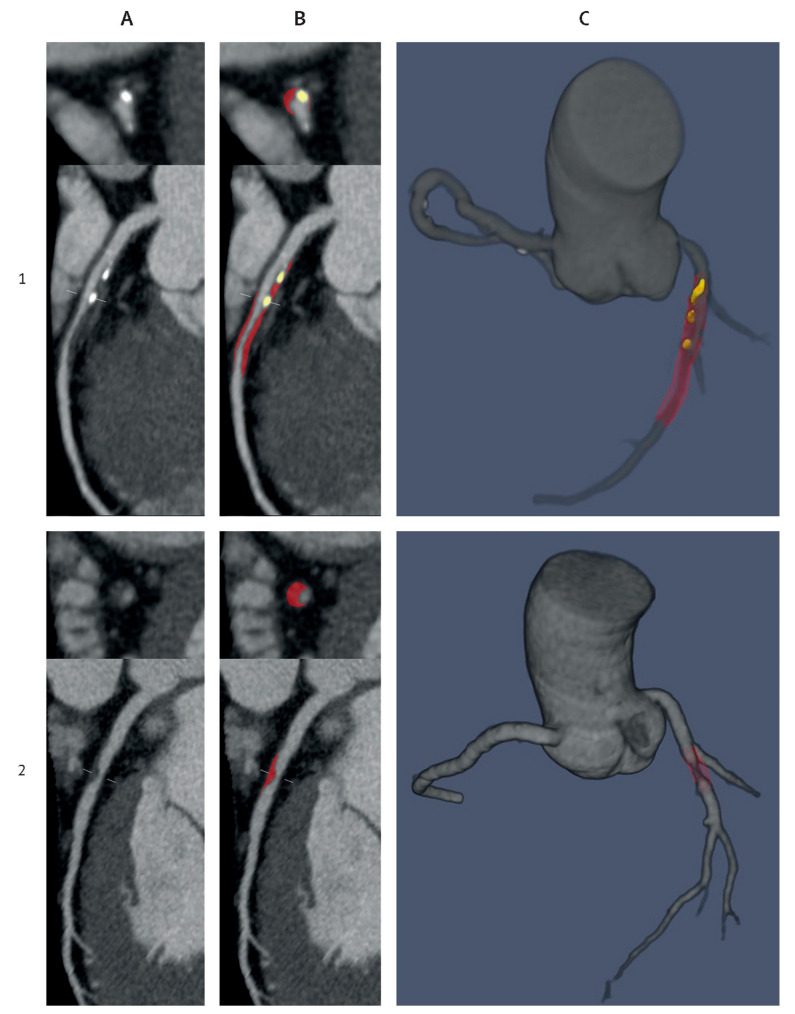
The use of deep learning for plaque segmentation. (**A**) Curved multi-planar reformation coronary CTA images showing lesions in the proximal-to-mid LAD (**1**) and the mid LAD (**2**). (**B**) Deep learning segmentation of calcified plaque (yellow) and non-calcified plaque (red). (**C**) Three-dimensionally rendered view of the coronary tree showing deep learning plaque segmentation in the individual analyzed segments. All lesions in each vessel were analyzed by deep learning and measurements summed on a per-patient level. CTA—computed tomography angiography; LAD—left anterior descending artery. Reprinted with permission under open access from Lin et al. [194].

Given the large number of images and coronary segments to review, resulting in a time-consuming task for human observers, DL has great potential to increase efficiency in interpreting coronary CTA images, with some reports documenting that DL similar or even better diagnostic performance when compared to manual observation [189,194,195,196,197,198,199]. Han and colleagues applied a DL model to 50 patients with CAD with AI-based coronary CTA, reducing time to image reconstructions by 85% and time to diagnose CAD by 80% [189]. AI-based coronary CTA and the traditional method of observation being carried out by expert observers have similar diagnostic value in identifying ≥ 50% coronary stenosis, with the corresponding sensitivity, specificity, positive predictive, and negative predictive values being 88% and 59%, 85% and 94%, 73% and 81%, 94% and 83%, respectively. The main limitation of using DL in coronary CTA is that most of the current studies are small proof-of-concept ones. This limitation has been addressed by a recent multi-center study involving nine cohorts at 11 international sites [194]. The novel DL model was trained on data from 921 patients (5045 lesions) for the automated segmentation of coronary plaques (Figure 29) before being applied to test 175 patients (1081 lesions), and another 50 patients (84 lesions) were assessed by intravascular ultrasound. The results showed excellent agreement and correlations between the DL model and expert readers in terms of measurements of plaque volume and lumen stenosis (intraclass correlation coefficient [ICC] 0.964 and 0.879, *p* < 0.001), as well as excellent agreement between DL and intravascular ultrasound in these two measurements (ICC 0.949 and 0.904). The mean time for per-patient plaque analysis was 5.65 s for the DL model and 25.66 min for the expert readers. DL-based measurements can predict the risk of future myocardial infarction; thus, DL has the potential to be implemented into routine cardiac CT workflows.

### 5.3. AI/ML/DL in Abdominal Aortic Aneurysm and Aortic Dissection

**Figure 30:**
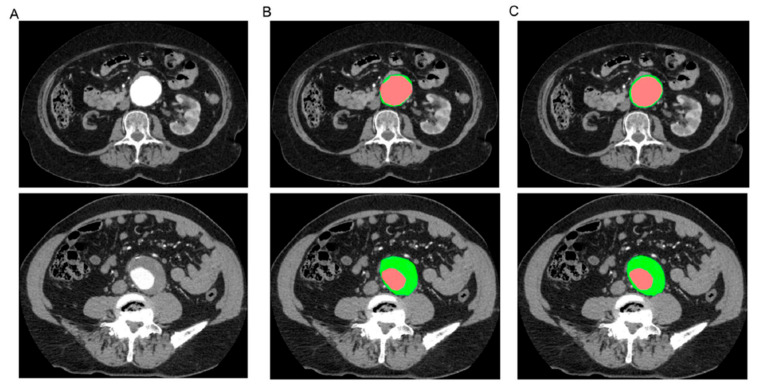
Representative images of the segmentation of the aortic lumen (in red) and the intraluminal thrombus (in green). (**A**) CT scan cross-sectional views of patients with infrarenal AAA. (**B**) Manual segmentation. (**C**) Automatic segmentation. Reprinted with permission under open access from Lareyre et al. [201].

Abdominal aortic aneurysm (AAA) is a common and life-threatening cardiovascular disease, and early diagnosis, especially the identification of the aneurysm growth and risk of rupture, plays an important role in improving the management of patients with AAA. The role of AI in the treatment of AAA patients has not been well explored. Raffort et al. conducted a comprehensive review about the usefulness of AI in AAA through an analysis of 34 studies [200]. Of these 34 studies, 15 were related to image segmentation and automation, 14 were related to the prediction and prognosis of patients, and 5 were related to AAA geometry and fluid dynamic analysis. Manual segmentation is time-consuming and also subject to inter-operator and intra-operator variations. With the use of AI, the mean segmentation time per patient was reduced to 7.4 min as opposed to 25–40 min per patient with the human manual segmentation method (Figure 30) [200,201]. This finding is similar to a recent study that showed the feasibility of using AI to screen for AAA [202]. Spinella et al. proposed a pipeline for the automatic segmentation of aortic lumen and thrombus and the calculation of maximal aortic aneurysm in 48 patients with AAA and 25 control patients. The average automatic lumen and thrombus segmentation time was 25 s and 63 s per scan, respectively, and the processing time for screening was 7.12 min. Their results showed the high value of their developed DL-based AAA screening method, which achieved 98% sensitivity, 96% specificity, and 97% accuracy in correctly classifying AAA and normal aortic dimensions.

Regarding the clinical value of using AI in prediction and prognosis, studies have demonstrated that AI is able to develop predictive morality scores in patients undergoing AAA repair and predict the risk of operative outcomes after endovascular aneurysm repair by discriminating patients at low risk of aortic complications from those at high risk of aortic complications [203,204,205]. In one specific study, a machine learning-based approach was shown to precisely characterize AAA geometry and calculate hemodynamic flow distribution at different cardiac cycle points, thus contributing to defining AAA risk rupture patterns [200].

Aortic dissection (AD) is another common cardiovascular disease often presenting as an emergency situation. Although contrast-enhanced CT is a routine modality for the diagnosis of AD with sensitivity and specificity values of more than 98% [1,3,6], AD can be missed upon non-contrast CT imaging due to non-specific symptoms. Hata and colleagues developed a deep learning algorithm for the detection of AD using the non-contrast-enhanced CT images of 170 patients [206]. Their developed DL algorithm showed high accuracy in terms of detecting AD on non-contrast CT images, with a sensitivity value of 91.8% and a specificity value of 88.2%, comparable to the performances of radiologists. This study shows the potential of using AI tools to reduce missed aortic dissection cases.

AI can also be applied to low- or ultra-low-dose aortic CTA to enhance image quality, despite the use of low-radiation doses and contrast medium doses. Zhou et al., in their recent study, reported the feasibility of using AI-based ultra-low-dose aortic CTA images in a prospective study that recruited 150 patients with suspected aortic disease [207]. Using an augmented cycle-consistent adversarial framework, the contrast medium dose was reduced by up to one-third of that from the low-dose contrast medium protocol. The AI-based ultra-low-dose protocol produced better quantitative images than the low-dose and ultra-low-dose protocols. There were no significant differences between these protocols regarding the diagnosis of aortic diseases.

### 5.4. AI/ML/DL in Pulmonary Artery Disease

CT pulmonary angiography (CTPA) is a standard imaging modality in diagnosing pulmonary embolisms (PEs) with high accuracy. However, the interpretation of CTPA images is time-consuming and requires radiologist expertise. Further, the use of CTPA in emergency departments has increased significantly over the last decades, and increased workloads and fatigue may lead to more diagnostic errors in emergency radiology [208]. Therefore, the use of an automatic PE detection method could assist radiologists’ decisions to ensure the rapid diagnosis of positive PE cases while avoiding mistakes.

**Figure 31:**
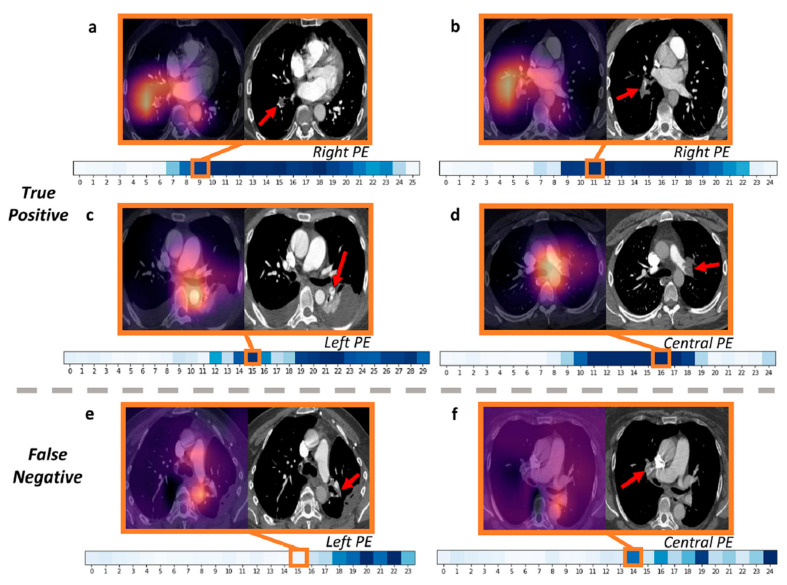
Interpretation with Grad-CAM and attention weights. True-positive (**a**–**d**) and false-negative (**e**,**f**) samples of Grad-CAM and original image for positional labels. For each sample, the processed CT image (**right**) and the corresponding attention-mapped image are paired (**left**). The red arrow points to the precise location of the PE identified by an experienced radiologist. The heatmap below shows the attention weights of all windows in the study containing the image above, while the orange square marks the exact window that includes the image. Darker colors in the heatmap illustrate larger attention weights. Reprinted with permission under open access from Ma et al. [211].

Studies have reported promising results regarding the application of DL models for the automatic detection of PEs based on CTPA images [209,210,211,212]. A recent systematic review and meta-analysis of using DL in the detection of PEs showed a pooled sensitivity of 88% and a specificity of 86% based on an analysis of five studies [209]. This indicates that further studies are required to validate the DL models to detect PEs based on large datasets of CTPA images. The Radiological Society of North America (RSNA) chose PE as its AI challenge in 2020, later publishing a public dataset of 12,195 annotated CTPA studies to encourage the development of DL models for PE detection [213]. Ma et al. applied their developed DL model to the RSNA Pulmonary Embolism Detection data for not only detecting PEs but also for predicting the position of PEs in pulmonary artery branches, predicting PE condition (acute or chronic), and analyzing specific features to suggest the presence of right heart strain [211]. Their model showed a sensitivity of 86% and a specificity of 85%, comparable to the performance of radiologists (the corresponding values ranged from 67 to 87% and 89 to 99%). Further, through the use of attention-weighted heatmaps and gradient-weighted class activation mapping (Grad-CAM), their model could predict PE existence and other properties associated with PE cases, which could assist PE diagnosis and management (Figure 31). A recent study by Grenier et al. also confirmed the high diagnostic value of using a DL model in PE detection [212]. The authors validated a commercially available AI model in 387 CTPA cases from multiple clinical sites (228 US clinical sites). The results showed a sensitivity of 91.4% and a specificity of 91.5%, along with an area under the receiver operating characteristic curve of 0.92.

**Figure 32:**
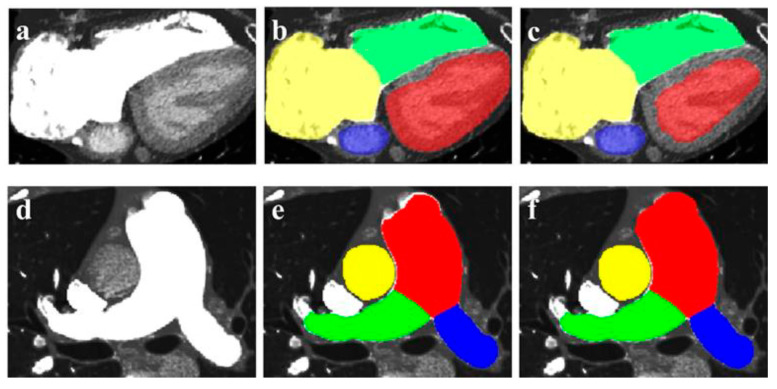
The performance of the proposed network framework. (**a**,**d**) The original images of the heart and pulmonary artery, respectively; (**b**,**e**) the segmentation outputs of nnU-Net; (**c**,**f**) the segmentation outputs of the proposed network framework. Segmented structures include right atrium (yellow), right ventricle (green), left atrium (blue), left ventricle (red), main pulmonary artery (red), right pulmonary artery (green) and left pulmonary artery (blue). Reprinted with permission under open access from Zhang et al. [214].

**Figure 33:**
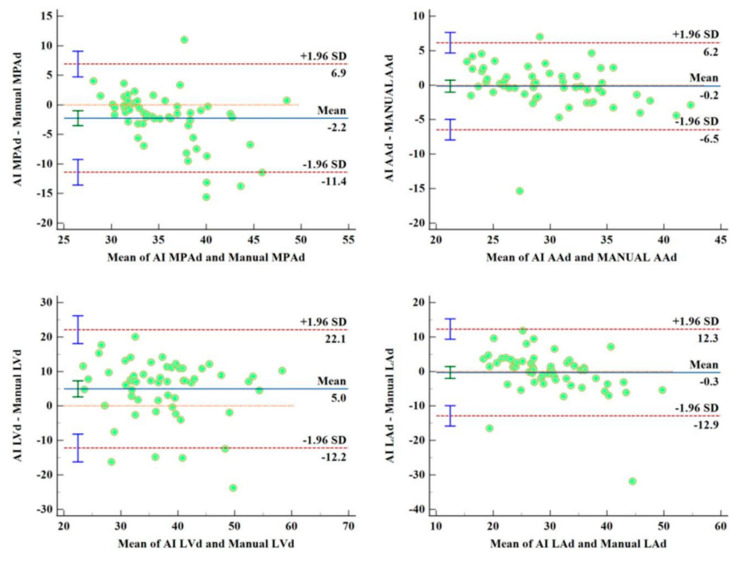
Bland–Altman analyses for features assessed by AI automatic and manual measurements show that the metrics measured by the automatic measurement method are in accordance with the ground-truth measured manually by experienced physicians. Reprinted with permission under open access from Zhang et al. [214].

AI is also used to assist the diagnosis of pulmonary hypertension based on CTPA [214]. Zhang et al. developed a fully automated CTPA-based framework through the segmentation of eight pulmonary and heart structures in 55 patients with pulmonary hypertension (Figure 32), followed by the AI-based automatic extraction of features associated with pulmonary artery pressure. The AI-based automatic extractions correlated well with the manual measurements (Figure 33). High consistency was found between the regression model and the gold standard in terms of the prediction of mean diastolic and systolic pulmonary artery pressure (*p* = 0.000). Their developed AI model contributes to the diagnosis and clinical management of patients with pulmonary hypertension.

## 6. Summary, Concluding Remarks, and Future Directions

Cardiovascular CT is playing an important role in the diagnosis of cardiovascular disease, and its role will continue to grow with further advancements in CT technology. The traditional reliance on the standard CT imaging approach has been significantly augmented with the use of recent technologies such as photon-counting CT, 3D printing, FFRCT, VR, AR, and MR, and AI. This recent progress has created great opportunities for incorporating these advanced technologies into education and clinical practice to achieve better outcomes.

The clinical value of CT has been further advanced with the recent emergence of photon-counting CT, which can be used to obtain images with superior spatial and contrast resolution. Despite being introduced into clinical practice very recently (in 2021), photon-counting CT represents the future of cardiovascular CT imaging and is set to revolutionize the current cardiovascular CT imaging approach, especially in the diagnostic assessment of cardiovascular disease. Since it is a very new technology that is not widely available, more promising results based on human studies are expected to be reported in the next few years.

**Figure 34:**
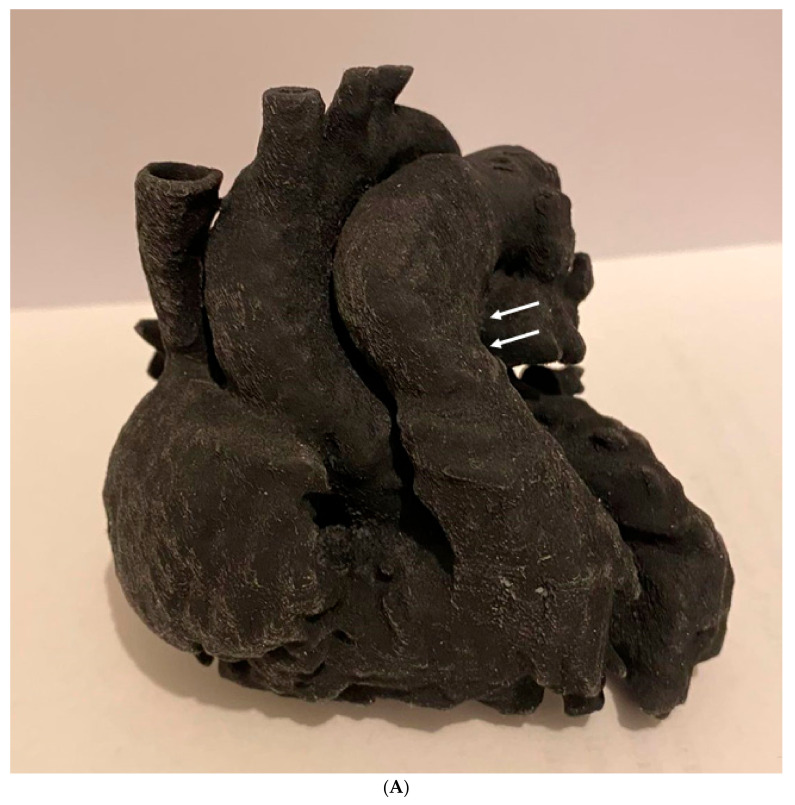

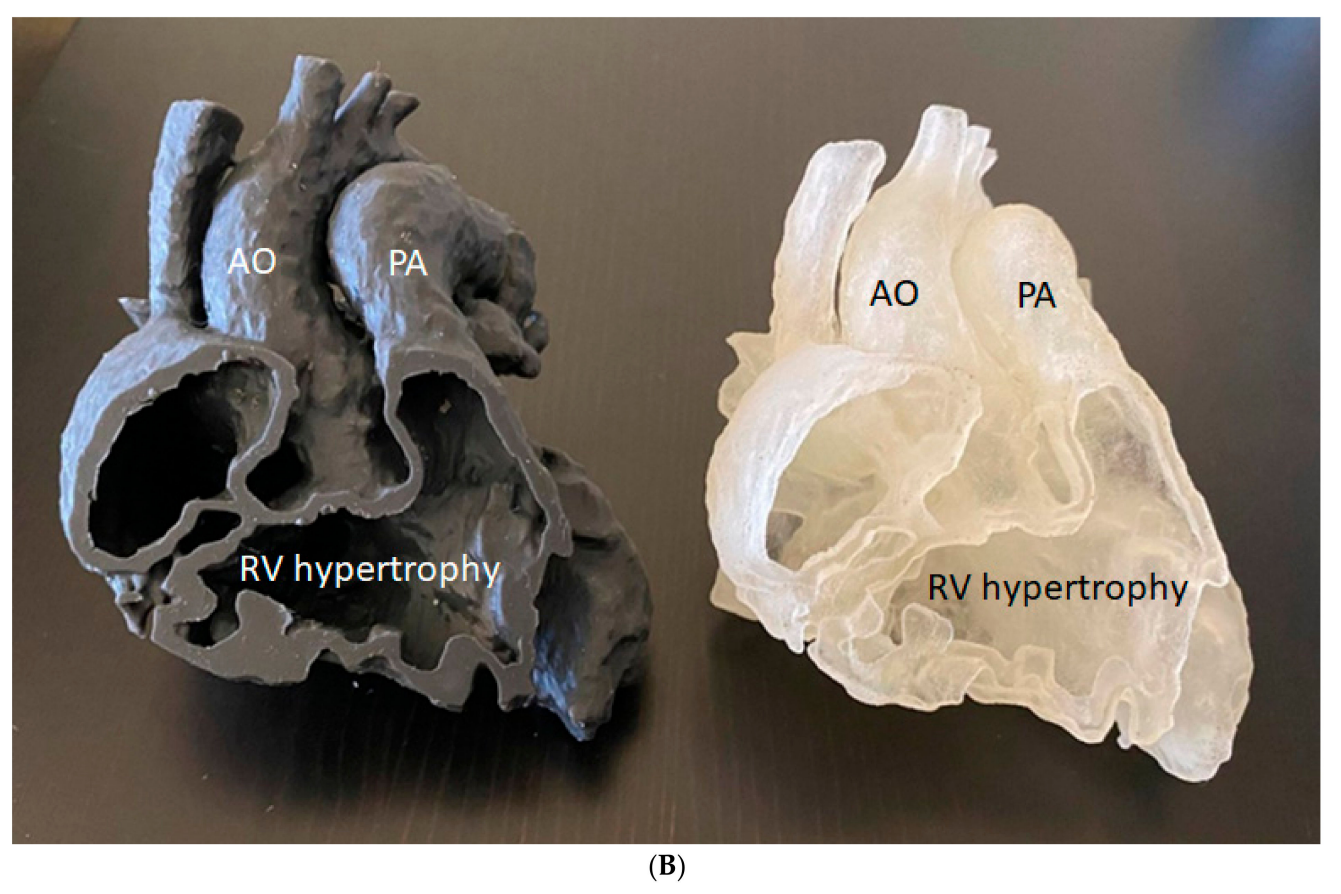
Three-dimensionally printed heart model of a patient with Tetralogy of Fallot. This model was printed based on cardiac CT images using Agilus30 material, and its tissue properties are similar to those of human heart tissues. The model was printed in one piece (**A**) and a two halves (**B**) to show the internal structures. The arrows refer to the pulmonary artery stenoses. AO—aorta, PA—pulmonary artery, RV—right ventricle. Reprinted with permission under open access from Sun [215].

The current applications of 3D printing technology in cardiovascular research are maturing, with more evidence available from multi-center or randomized controlled trials being developed. Three-dimensionally printed models are highly accurate and reliable when it comes to replicating both cardiovascular anatomy and pathology, thus serving as a useful tool for medical education, surgical planning, and the simulation of challenging cardiovascular procedures, guiding intraoperative surgeries to improve patient outcomes. Three-dimensionally printed models improve communication between clinicians and patients, as well as communication between clinical colleagues. Owing to the development of more 3D printing materials that are able to simulate cardiovascular tissue properties (Figure 34) [215], more flexible and realistic 3D models could be printed in the future to further advance the efficacy of using 3D printing technology in cardiovascular disease by, for example, carrying out simulations of cardiovascular hemodynamics related to cardiovascular disease. Clinical studies, in particular longitudinal follow-ups on the impact of using 3D-printed models on patients’ clinical outcomes and cost-effectiveness, are still lacking, and these topics should remain the focus of further studies. Guidelines for appropriate criteria need to be developed regarding the use of 3D printing in cardiovascular disease, as such guidelines would provide guidance for clinical practice. 

The clinical value of FFRCT has been validated by several multi-center randomized controlled studies and many single-site studies, and its role will continue to grow with the increasing use of DL algorithms in the medical domain. The main barrier to implementing FFRCT in daily cardiology practice lies in the fact that most of the data analyses have been performed at off-site workstations, although on-site image processing and analyses are available, as evidenced by the TARGET trial. With the increasing prevalence of DL models and widespread use of AI in clinical practice, FFRCT will be implemented into diagnostic approaches to guide the revascularization of patients with coronary artery disease, leading to improvements in the utilization of healthcare resources.

VR, AR, and, more recently, MR are showing great promise, and they are set to complement traditional visualizations and assist healthcare providers and patients with cardiovascular disease. However, their applications in current practice are still at an early stage of development due to several limitations. First, the real-time integration of cardiovascular CT imaging in a VR/AR environment is challenging, as most of the current studies have applied segmented datasets to VR/AR/MR glasses or head-mounted devices. Second, a critical aspect regarding the use of AR and MR in surgical planning or guiding surgical procedures is to balance AR/MR with the real-world environment and add digital elements to the field of view to achieve the harmonization of data flow and interfaces. Third, ethical considerations need to be considered, as the main goal of using VR/AR/MR should focus on enhancing the patient–provider relationship. Hepatic feedback (the tactile experience that is available with 3D-printed physical models) is lacking in VR/AR/MR techniques, so this needs to be addressed in future research. 

**Figure 35:**
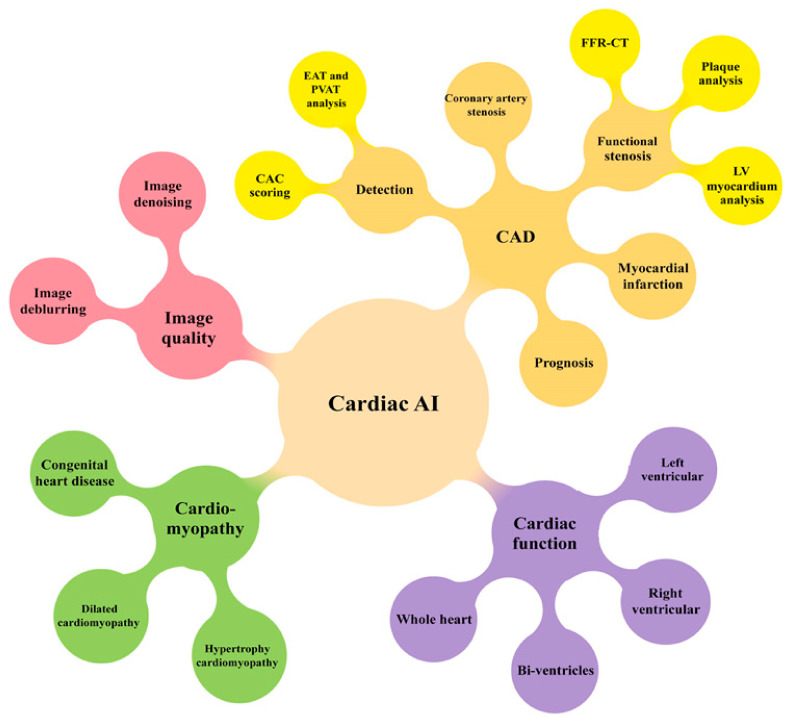
The applications of artificial intelligence in clinical cardiology practice. CAC—coronary calcium score, CAD—coronary artery disease, EAT—epicardial adipose tissue, PVAT—perivascular adipose tissue, LV—left ventricle. Reprinted with permission under open access from Jiang et al. [216].

The widespread use of AI in medicine and the application of AI in cardiovascular disease is inevitable, and clinicians must be aware of pitfalls when applying this rapidly evolving technology to their practice. Figure 35 is a summary of AI applications in cardiology practice [216]. Given the wide range of AI algorithms available, the input data used for training purposes must be examined to ensure high data quality. AI model performance must be examined to guarantee that findings are robust, and external validation is also an important consideration. Medical graduates and clinicians’ skills and confidence in managing AI applications need to be improved, as this will have a direct impact on using AI in clinical practice. Ethical issues related to the sharing of healthcare data and legal challenges should be addressed, and AI should be included in the medical curriculum and professional education. Collaboration among a multi-disciplinary team consisting of computer scientists, clinicians, clinical investigators, academic researchers, and other users is essential for identifying the best approach and data sources to achieve the goal of delivering personalized treatment in cardiovascular disease cases. The incorporation of these technologies beyond standard cardiovascular CT into routine diagnostic workflows and clinical decision making is expected to occur soon.

## Figures and Tables

**Table 1:** Benefits of using photon-counting detectors and their impacts on cardiovascular applications. Reprinted with permission under open access from Cademartiri et al. [22].

Benefits of Photon-Counting Detectors	Potential Cardiovascular Applications
Higher spatial resolution	Stent imaging
	Coronary lumen evaluation
	Atherosclerotic plaque imaging
	Coronary artery calcium scoring
	Aortic valve calcification score
Improved iodine signal	Coronary lumen evaluation
	Stent imaging
Multi-energy acquisition	Coronary lumen evaluation
	Atherosclerotic plaque imaging
	Dose reduction
	Coronary artery calcium scoring
	Aortic valve calcification score
Energy binning	Stent imaging
	Atherosclerotic plaque imaging
	Dose reduction
	Myocardial tissue characterization.
Artifact reduction	Coronary lumen evaluation
	Stent imaging
	Atherosclerotic plaque imaging

## Data Availability

Not applicable.

## References

[1] Gulsin G.S., McVeigh N., Leipsic J.A., Dodd J.D. (2021). Cardiovascular CT and MRI in 2020: Review of key articles. Radiology.

[2] Sayed A., Munir M., Bahbah E.I. (2021). Aortic Dissection: A Review of the Pathophysiology, Management and Prospective Advances. Curr. Cardiol. Rev..

[3] Abbas A., Brown I., Peebles C., Harden S., Shambrook J. (2014). The role of multidetector-row CT in the diagnosis, classification and management of acute aortic syndrome. Br. J. Radiol..

[4] Seitun S., Clemente A., Maffei E., Toia P., La Grutta L., Cademartiri F. (2020). Prognostic value of cardiac CT. Radiol. Medica.

[5] Sun Z. (2012). Cardiac CT imaging in coronary artery disease: Current status and future directions. Quant. Imaging Med. Surg..

[6] Al’Aref S.J., Min J.K. (2019). Cardiac CT: Current practice and emerging applications. Heart.

[7] Mayo J., Thakur Y. (2013). Pulmonary CT Angiography as First-Line Imaging for PE: Image Quality and Radiation Dose Considerations. AJR Am. J. Roentgenol..

[8] Mayo J., Thakur Y. (2014). Acute Pulmonary Embolism: From Morphology to Function. Semin. Respir. Crit. Care Med..

[9] Corballis N., Tsampasian V., Merinopoulis I., Gunawardena T., Bhalraam U., Eccleshall S., Dweck M.R., Vassiliou V. (2023). CT angiography compared to invasive angiography for stable coronary disease as predictors of major adverse cardiovascular events—A systematic review and meta-analysis. Heart Lung.

[10] Counseller Q., Aboelkassem Y. (2023). Recent technologies in cardiac imaging. Front. Med. Technol..

[11] Newby D., Williams M., Hunter A., Pawade T., Shah A., Flapan A., Forbes J., Hargreaves A., Stephen L., Lewis S. (2015). CT coronary angiography in patients with suspected angina due to coronary heart disease (SCOT-HEART): An open-label, parallel-group, multicentre trial. Lancet.

[12] Maurovich-Horvat P., Bosserdt M., Kofoed K.F., Rieckmann N., Benedek T., Donnelly P., Rodriguez-Palomares J., Erglis A., Štěchovský C., Šakalyte G. (2022). CT or invasive coronary angiography in stable chest pain. N. Engl. J. Med..

[13] Sun Z. (2006). Diagnostic Accuracy of Multislice CT Angiography in Peripheral Arterial Disease. J. Vasc. Interv. Radiol..

[14] Walls M.C., Thavendiranathan P., Rajagopalan S. (2011). Advances in CT angiography for peripheral arterial disease. Cardiol. Clin..

[15] Kim J.W., Choo K.S., Jeon U.B., Kim T.U., Hwang J.Y., Yeom J.A., Jeong H.S., Choi Y.Y., Nam K.J., Kim C.W. (2016). Diagnostic performance and radiation dose of lower extremity CT angiography using a 128-slice dual source CT at 80 kVp and high pitch. Acta. Radiol..

[16] Kalisz K., Halliburton S., Abbara S., Leipsic J.A., Albrecht M.H., Schoepf U.J., Rajiah P. (2017). Update on cardiovascular applications of multienergy CT. Radiographics.

[17] Machida H., Tanaka I., Fukui R., Shen Y., Ishikawa T., Tate E., Ueno E. (2016). Dual-energy spectral CT: Various clinical vascular applications. Radiographics.

[18] Litmanovich D.E., Tack D.M., Shahrzad M., Bankier A.A. (2014). Dose reduction in cardiothoracic CT: Review of currently available methods. Radiographics.

[19] Usai M.V., Gerwing M., Gottschalk A., Sporns P., Heindel W., Oberhuber A., Wildgruber M., Köhler M. (2019). Intra-arterial catheter-directed CT angiography for assessment of endovascular aortic aneurysm repair. PLoS ONE.

[20] Schuijf J.D., Lima J.A.C., Boedeker K.L., Takagi H., Tanaka R., Yoshioka K., Arbab-Zadeh A. (2022). CT imaging with ultra-high-resolution: Opportunities for cardiovascular imaging in clinical practice. J. Cardiovasc. Comput. Tomogr..

[21] Tortora M., Gemini L., D’Iglio I., Ugga L., Spadarella G., Cuocolo R. (2022). Spectral Photon-Counting Computed Tomography: A Review on Technical Principles and Clinical Applications. J. Imaging.

[22] Cademartiri F., Meloni A., Pistoia L., Degiorgi G., Clemente A., Gori C.D., Positano V., Celi S., Berti S., Emdin M. (2023). Dual-Source Photon-Counting Computed Tomography&mdash; Part I: Clinical Overview of Cardiac CT and Coronary CT Angiography Applications. J. Clin. Med..

[23] Si-Mohamed S.A., Boccalini S., Lacombe H., Diaw A., Varasteh M., Rodesch P.-A., Dessouky R., Villien M., Tatard-Leitman V., Bochaton T. (2022). Coronary CT angiography with photon-counting CT: First-in-human results. Radiology.

[24] Flohr T., Schmidt B., Ulzheimer S., Alkadhi H. (2023). Cardiac imaging with photon counting CT. Br. J. Radiol..

[25] Allmendinger T., Nowak T., Flohr T., Klotz E., Hagenauer J., Alkadhi H., Schmidt B. (2022). Photon-Counting Detector CT-Based Vascular Calcium Removal Algorithm: Assessment Using a Cardiac Motion Phantom. Investig. Radiol..

[26] Boccalini S., Si-Mohamed S.A., Lacombe H., Diaw A., Varasteh M., Rodesch P.A., Villien M., Sigovan M., Dessouky R., Coulon P. (2022). First in-Human Results of Computed Tomography Angiography for Coronary Stent Assessment with a Spectral Photon Counting Computed Tomography. Investig. Radiol..

[27] Koons E., VanMeter P., Rajendran K., Yu L., McCollough C., Leng S. (2022). Improved quantification of coronary artery luminal stenosis in the presence of heavy calcifications using photon-counting detector CT. Proc. SPIE. Int. Soc. Opt. Eng..

[28] Meloni A., Frijia F., Panetta D., Degiorgi G., De Gori C., Maffei E., Clemente A., Positano V., Cademartiri F. (2023). Photon-counting computed tomography (pcct): Technical background and cardio-vascular applications. Diagnostics.

[29] Bech G.J.W., De Bruyne B., Pijls N.H., De Muinck E.D., Hoorntje J.C., Escaned J., Stella P.R., Boersma E., Bartunek J., Koolen J.J. (2001). Fractional flow reserve to determine the appropriateness of angioplasty in moderate coronary stenosis: A randomized trial. Circulation.

[30] Tavoosi A., Kadoya Y., Chong A.Y., Small G.R., Chow B.J.W. (2023). Utility of FFRCT in Patients with Chest Pain. Curr. Atheroscler. Rep..

[31] Chen J., Wetzel L.H., Pope K.L., Meek L.J., Rosamond T., Walker C.M. (2020). FFRCT: Current Status. AJR Am. J. Roentgenol..

[32] Ihdayhid A.R., Norgaard B.L., Gaur S., Leipsic J., Nerlekar N., Osawa K., Miyoshi T., Jensen J.M., Kimura T., Shiomi H. (2019). Prognostic value and risk continuum of noninvasive fractional flow reserve derived from coronary CT angiography. Radiology.

[33] Kawaji T., Shiomi H., Morishita H., Morimoto T., Taylor C.A., Kanao S., Koizumi K., Kozawa S., Morihiro K., Watanabe H. (2017). Feasibility and diagnostic performance of fractional flow reserve measurement derived from coronary computed tomography angiography in real clinical practice. Int. J. Cardiovasc. Imaging.

[34] Nørgaard B.L., Jensen J.M., Blanke P., Sand N.P., Rabbat M., Leipsic J. (2017). Coronary CT Angiography Derived Fractional Flow Reserve: The Game Changer in Noninvasive Testing. Curr. Cardiol. Rep..

[35] Xu L., Sun Z., Fan Z. (2015). Noninvasive Physiologic Assessment of Coronary Stenoses Using Cardiac CT. BioMed Res. Int..

[36] Gao X., Wang R., Sun Z., Zhang H., Bo K., Xue X., Yang J., Xu L. (2023). A Novel CT Perfusion-Based Fractional Flow Reserve Algorithm for Detecting Coronary Artery Disease. J. Clin. Med..

[37] Fairbairn T.A., Nieman K., Akasaka T., Nørgaard B.L., Berman D.S., Raff G., Hurwitz-Koweek L.M., Pontone G., Kawasaki T., Sand N.P. (2018). Real-world clinical utility and impact on clinical decision-making of coronary computed tomography angiography-derived fractional flow reserve: Lessons from the ADVANCE Registry. Eur. Heart J..

[38] Xue X., Liu X., Gao Z., Wang R., Xu L., Ghista D., Zhang H. (2023). Personalized coronary blood flow model based on CT perfusion to non-invasively calculate fractional flow reserve. Comput. Methods Appl. Mech. Eng..

[39] Donnelly P.M., Kolossváry M., Karády J., Ball P.A., Kelly S., Fitzsimons D., Spence M.S., Celeng C., Horváth T., Szilveszter B. (2018). Experience with an on-Site Coronary Computed Tomography-Derived Fractional Flow Reserve Algorithm for the Assessment of Intermediate Coronary Stenoses. Am. J. Cardiol..

[40] Yang D.H., Kim Y.-H., Roh J.H., Kang J.-W., Ahn J.-M., Kweon J., Lee J.B., Choi S.H., Shin E.-S., Park D.-W. (2016). Diagnostic performance of on-site CT-derived fractional flow reserve versus CT perfusion. Eur. Heart J.—Cardiovasc. Imaging.

[41] Koo B.-K., Erglis A., Doh J.-H., David V.D., Jegere S., Kim H.-S., Dunning A., DeFrance T., Lansky A., Leipsic J. (2011). Diagnosis of Ischemia-Causing Coronary Stenoses by Noninvasive Fractional Flow Reserve Computed from Coronary Computed Tomographic Angiograms. J. Am. Coll. Cardiol..

[42] Yoon Y.E., Choi J.-H., Kim J.-H., Park K.-W., Doh J.-H., Kim Y.-J., Koo B.-K., Min J.K., Erglis A., Gwon H.-C. (2012). Noninvasive Diagnosis of Ischemia-Causing Coronary Stenosis Using CT Angiography. JACC Cardiovasc. Imaging.

[43] Nørgaard B.L., Leipsic J., Gaur S., Seneviratne S., Ko B.S., Ito H., Jensen J.M., Mauri L., De Bruyne B., Bezerra H. (2014). Diagnostic Performance of Noninvasive Fractional Flow Reserve Derived from Coronary Computed Tomography Angiography in Suspected Coronary Artery Disease. J. Am. Coll. Cardiol..

[44] Colleran R., Douglas P.S., Hadamitzky M., Gutberlet M., Lehmkuhl L., Foldyna B., Woinke M., Hink U., Nadjiri J., Wilk A. (2017). An FFRCT diagnostic strategy versus usual care in patients with suspected coronary artery disease planned for invasive coronary angiography at German sites: One-year results of a subgroup analysis of the PLATFORM (Prospective Longitudinal Trial of FFRCT: Outcome and Resource Impacts) study. Open Heart.

[45] Douglas P.S., Pontone G., Hlatky M.A., Patel M.R., Norgaard B.L., Byrne R.A., Curzen N., Purcell I., Gutberlet M., Rioufol G. (2015). Clinical outcomes of fractional flow reserve by computed tomographic angiography-guided diagnostic strategies vs. usual care in patients with suspected coronary artery disease: The prospective longitudinal trial of FFRCT: Outcome and resource impacts study. Eur. Heart J..

[46] Patel M.R., Nørgaard B.L., Fairbairn T.A., Nieman K., Akasaka T., Berman D.S., Raff G.L., Koweek L.M.H., Pontone G., Kawasaki T. (2020). 1-Year Impact on Medical Practice and Clinical Outcomes of FFRCT. JACC Cardiovasc. Imaging.

[47] Curzen N.P., Nolan J., Zaman A.G., Nørgaard B.L., Rajani R. (2016). Does the Routine Availability of CT–Derived FFR Influence Management of Patients with Stable Chest Pain Compared to CT Angiography Alone?: The FFRCT RIPCORD Study. JACC Cardiovasc. Imaging.

[48] Curzen N., Nicholas Z., Stuart B., Wilding S., Hill K., Shambrook J., Eminton Z., Ball D., Barrett C., Johnson L. (2021). Fractional flow reserve derived from computed tomography coronary angiography in the assessment and management of stable chest pain: The FORECAST randomized trial. Eur. Heart J..

[49] Giannopoulos A.A., Steigner M.L., George E., Barile M., Hunsaker A.R., Rybicki F.J., Mitsouras D. (2016). Cardiothoracic Applications of 3-dimensional Printing. J. Thorac. Imaging.

[50] Costello J.P., Olivieri L.J., Krieger A., Thabit O., Marshall M.B., Yoo S.-J., Kim P.C., Jonas R.A., Nath D.S. (2014). Utilizing Three-Dimensional Printing Technology to Assess the Feasibility of High-Fidelity Synthetic Ventricular Septal Defect Models for Simulation in Medical Education. World. J. Pediatr. Congenit. Heart Surg..

[51] Costello J.P., Olivieri L.J., Su L., Krieger A., Alfares F., Thabit O., Marshall M.B., Yoo S.J., Kim P.C., Jonas R.A. (2015). Incorporating three-dimensional printing into a simulation-based congenital heart disease and critical care training curriculum for resident physicians. Congenit. Heart Dis..

[52] Sun Z., Lau I., Wong Y.H., Yeong C.H. (2019). Personalized three-dimensional printed models in congenital heart disease. J. Clin. Med..

[53] Sun Z., Shen-Yuan L. (2017). A systematic review of 3-D printing in cardiovascular and cerebrovascular diseases. Anatol. J. Cardiol..

[54] Shabbak A., Masoumkhani F., Fallah A., Amani-Beni R., Mohammadpour H., Shahbazi T., Bakhshi A. (2024). 3D printing for cardiovascular surgery and intervention: A review article. Curr. Probl. Cardiol..

[55] Lau I., Sun Z. (2018). Three-dimensional printing in congenital heart disease: A systematic review. J. Med. Radiat. Sci..

[56] Verghi E., Catanese V., Nenna A., Montelione N., Mastroianni C., Lusini M., Stilo F., Chello M. (2021). 3D printing in cardiovascular disease: Current appplications an future perspectives. Surg. Technol. Int..

[57] Lau I.W.W., Sun Z. (2019). Dimensional Accuracy and Clinical Value of 3D Printed Models in Congenital Heart Disease: A Systematic Review and Meta-Analysis. J. Clin. Med..

[58] Sun Z. (2020). Clinical Applications of Patient-Specific 3D Printed Models in Cardiovascular Disease: Current Status and Future Directions. Biomolecules.

[59] Gómez-Ciriza G., Gómez-Cía T., Rivas-González J.A., Forte M.N.V., Valverde I. (2021). Affordable three-dimensional printed heart models. Front. Cardiovasc. Med..

[60] Moore R.A., Riggs K.W., Kourtidou S., Schneider K., Szugye N., Troja W., D’Souza G., Rattan M., Bryant R., Taylor M.D. (2018). Three-dimensional printing and virtual surgery for congenital heart procedural planning. Birth Defects Res..

[61] Garas M., Vaccarezza M., Newland G., McVay-Doornbusch K., Hasani J. (2018). 3D-Printed specimens as a valuable tool in anatomy education: A pilot study. Ann. Anat..

[62] Anwar S., Rockefeller T., Raptis D.A., Woodard P.K., Eghtesady P. (2018). 3D Printing Provides a Precise Approach in the Treatment of Tetralogy of Fallot, Pulmonary Atresia with Major Aortopulmonary Collateral Arteries. Curr. Treat. Options Cardiovasc. Med..

[63] Loke Y.-H., Harahsheh A.S., Krieger A., Olivieri L.J. (2017). Usage of 3D models of tetralogy of Fallot for medical education: Impact on learning congenital heart disease. BMC Med. Educ..

[64] Bouraghi H., Mohammadpour A., Khodaveisi T., Ghazisaeedi M., Saeedi S., Familgarosian S. (2023). Virtual Reality and Cardiac Diseases: A Systematic Review of Applications and Effects. J. Healthc. Eng..

[65] Stephenson N., Pushparajah K., Wheeler G., Deng S., Schnabel J.A., Simpson J.M. (2023). Extended reality for procedural planning and guidance in structural heart disease—A review of the state-of-the-art. Int. J. Cardiovasc. Imaging.

[66] Maresky H., Oikonomou A., Ali I., Ditkofsky N., Pakkal M., Ballyk B. (2019). Virtual reality and cardiac anatomy: Exploring immersive three-dimensional cardiac imaging, a pilot study in undergraduate medical anatomy education. Clin. Anat..

[67] Barteit S., Lanfermann L., Bärnighausen T., Neuhann F., Beiersmann C. (2021). Augmented, mixed, and virtual reality-based head-mounted devices for medical education: Systematic review. JMIR Serious Games.

[68] Dhar P., Rocks T., Samarasinghe R.M., Stephenson G., Smith C. (2021). Augmented reality in medical education: Students’ experiences and learning outcomes. Med. Educ. Online.

[69] Dilsizian S.E., Siegel E.L. (2013). Artificial Intelligence in Medicine and Cardiac Imaging: Harnessing Big Data and Advanced Computing to Provide Personalized Medical Diagnosis and Treatment. Curr. Cardiol. Rep..

[70] Nakanishi R., Sankaran S., Grady L., Malpeso J., Yousfi R., Osawa K., Ceponiene I., Nazarat N., Rahmani S., Kissel K. (2018). Automated estimation of image quality for coronary computed tomographic angiography using machine learning. Eur. Radiol..

[71] Wang D.D., Qian Z., Vukicevic M., Engelhardt S., Kheradvar A., Zhang C., Stephen H.L., Verjans J., Comaniciu D., O’Neill William W. (2021). 3D Printing, Computational Modeling, and Artificial Intelligence for Structural Heart Disease. JACC Cardiovasc. Imaging.

[72] Krittanawong C., Zhang H., Wang Z., Aydar M., Kitai T. (2017). Artificial Intelligence in Precision Cardiovascular Medicine. J. Am. Coll. Cardiol..

[73] Lopez-Jimenez F., Attia Z., Arruda-Olson A.M., Carter R., Chareonthaitawee P., Jouni H., Kapa S., Lerman A., Luong C., Medina-Inojosa J.R. (2020). Artificial Intelligence in Cardiology: Present and Future. Mayo Clin. Proc..

[74] Park M.J., Jung J.I., Choi Y.-S., Ann S.H., Youn H.-J., Jeon G.N., Choi H.C. (2011). Coronary CT angiography in patients with high calcium score: Evaluation of plaque characteristics and diagnostic accuracy. Int. J. Cardiovasc. Imaging.

[75] Vavere A.L., Arbab-Zadeh A., Rochitte C.E., Dewey M., Niinuma H., Gottlieb I., Clouse M.E., Bush D.E., Hoe J.W., de Roos A. (2011). Coronary artery stenoses: Accuracy of 64-detector row CT angiography in segments with mild, moderate, or severe calcification—A subanalysis of the CORE-64 trial. Radiology.

[76] Chen C.-C., Chen C.-C., Hsieh I.C., Liu Y.-C., Liu C.-Y., Chan T., Wen M.-S., Wan Y.-L. (2011). The effect of calcium score on the diagnostic accuracy of coronary computed tomography angiography. Int. J. Cardiovasc. Imaging.

[77] Meng L., Cui L., Cheng Y., Wu X., Tang Y., Wang Y., Xu F. (2009). Effect of heart rate and coronary calcification on the diagnostic accuracy of the dual-source CT coronary angiography in patients with suspected coronary artery disease. Korean J. Radiol..

[78] Meijs M.F.L., Meijboom W.B., Prokop M., Mollet N.R., van Mieghem C.A.G., Doevendans P.A., de Feyter P.J., Cramer M.J. (2009). Is there a role for CT coronary angiography in patients with symptomatic angina? Effect of coronary calcium score on identification of stenosis. Int. J. Cardiovasc. Imaging.

[79] Sun Z., Ng C.K. (2015). High calcium scores in coronary CT angiography: Effects of image post-processing on visualization and measurement of coronary lumen diameter. J. Med. Imaging Health Inform..

[80] Knuuti J., Wijns W., Saraste A., Capodanno D., Barbato E., Funck-Brentano C., Prescott E., Storey R.F., Deaton C., Cuisset T. (2019). 2019 ESC Guidelines for the diagnosis and management of chronic coronary syndromes: The Task Force for the diagnosis and management of chronic coronary syndromes of the European Society of Cardiology (ESC). Eur. Heart J..

[81] Sun Z., Ng C.K.C., Xu L., Fan Z., Lei J. (2015). Coronary CT Angiography in Heavily Calcified Coronary Arteries: Improvement of Coronary Lumen Visualization and Coronary Stenosis Assessment with Image Postprocessing Methods. Medicine.

[82] Funabashi N., Irie R., Aiba M., Morimoto R., Kabashima T., Fujii S., Uehara M., Ozawa K., Takaoka H., Kobayashi Y. (2013). Adaptive-Iterative-Dose-Reduction 3D with multisector-reconstruction method in 320-slice CT may maintain accurate-measurement of the Agatston-calcium-score of severe-calcification even at higher pulsating-beats and low tube-current in vitro. Int. J. Cardiol..

[83] van Osch J.A.C., Mouden M., van Dalen J.A., Timmer J.R., Reiffers S., Knollema S., Greuter M.J.W., Ottervanger J.P., Jager P.L. (2014). Influence of iterative image reconstruction on CT-based calcium score measurements. Int. J. Cardiovasc. Imaging.

[84] Renker M., Nance J.W., Schoepf U.J., O’Brien T.X., Zwerner P.L., Meyer M., Kerl J.M., Bauer R.W., Fink C., Vogl T.J. (2011). Evaluation of heavily calcified vessels with coronary CT angiography: Comparison of iterative and filtered back projection image reconstruction. Radiology.

[85] Tanaka R., Yoshioka K., Muranaka K., Chiba T., Ueda T., Sasaki T., Fusazaki T., Ehara S. (2013). Improved evaluation of calcified segments on coronary CT angiography: A feasibility study of coronary calcium subtraction. Int. J. Cardiovasc. Imaging.

[86] Pontone G., Bertella E., Mushtaq S., Loguercio M., Cortinovis S., Baggiano A., Conte E., Annoni A., Formenti A., Beltrama V. (2014). Coronary artery disease: Diagnostic accuracy of CT coronary angiography—A comparison of high and standard spatial resolution scanning. Radiology.

[87] Latina J., Shabani M., Kapoor K., Whelton S.P., Trost J.C., Sesso J., Demehri S., Mahesh M., Lima J.A., Arbab-Zadeh A. (2021). Ultra-high-resolution coronary CT angiography for assessment of patients with severe coronary artery calcification: Initial experience. Radiol. Cardiothorac. Imaging.

[88] Sun Z., Ng C.K. (2022). Artificial intelligence (enhanced super-resolution generative adversarial network) for calcium deblooming in coronary computed tomography angiography: A feasibility study. Diagnostics.

[89] Sun Z., Ng C.K. (2022). Finetuned super-resolution generative adversarial network (artificial intelligence) model for calcium deblooming in coronary computed tomography angiography. J. Pers. Med..

[90] Ng C.K., Sun Z., Jansen S. (2023). Comparison of performance of micro-computed tomography (Micro-CT) and synchrotron radiation CT in assessing coronary stenosis caused by calcified plaques in coronary artery phantoms. J. Vasc. Dis..

[91] Sun Z., Xu L., Fan Z. (2016). Coronary CT angiography in calcified coronary plaques: Comparison of diagnostic accuracy between bifurcation angle measurement and coronary lumen assessment for diagnosing significant coronary stenosis. Int. J. Cardiol..

[92] Sun Z. (2013). Coronary CT angiography in coronary artery disease: Correlation between virtual intravascular endoscopic appearances and left bifurcation angulation and coronary plaques. BioMed Res. Int..

[93] Xu L., Sun Z. (2015). Coronary CT angiography evaluation of calcified coronary plaques by measurement of left coronary bifurcation angle. Int. J. Cardiol..

[94] Mergen V., Eberhard M., Manka R., Euler A., Alkadhi H. (2022). First in-human quantitative plaque characterization with ultra-high resolution coronary photon-counting CT angiography. Front. Cardiovasc. Med..

[95] Wolf E.V., Halfmann M.C., Schoepf U.J., Zsarnoczay E., Fink N., Griffith J.P., Aquino G.J., Willemink M.J., O’Doherty J., Hell M.M. (2023). Intra-individual comparison of coronary calcium scoring between photon counting detector-and energy integrating detector-CT: Effects on risk reclassification. Front. Cardiovasc. Med..

[96] Soschynski M., Hagen F., Baumann S., Hagar M.T., Weiss J., Krauss T., Schlett C.L., von zur Mühlen C., Bamberg F., Nikolaou K. (2022). High Temporal Resolution Dual-Source Photon-Counting CT for Coronary Artery Disease: Initial Multicenter Clinical Experience. J. Clin. Med..

[97] Karsenty C., Guitarte A., Dulac Y., Briot J., Hascoet S., Vincent R., Delepaul B., Vignaud P., Djeddai C., Hadeed K. (2021). The usefulness of 3D printed heart models for medical student education in congenital heart disease. BMC Med. Educ..

[98] Lim K.H.A., Loo Z.Y., Goldie S.J., Adams J.W., McMenamin P.G. (2016). Use of 3D printed models in medical education: A randomized control trial comparing 3D prints versus cadaveric materials for learning external cardiac anatomy. Anat. Sci. Educ..

[99] Su W., Xiao Y., He S., Huang P., Deng X. (2018). Three-dimensional printing models in congenital heart disease education for medical students: A controlled comparative study. BMC Med. Educ..

[100] Smith C.F., Tollemache N., Covill D., Johnston M. (2018). Take away body parts! An investigation into the use of 3D-printed anatomical models in undergraduate anatomy education. Anat. Sci. Educ..

[101] Yi X., Ding C., Xu H., Huang T., Kang D., Wang D. (2019). Three-Dimensional Printed Models in Anatomy Education of the Ventricular System: A Randomized Controlled Study. World Neurosurg..

[102] Mogali S.R., Chandrasekaran R., Radzi S., Peh Z.K., Tan G.J.S., Rajalingam P., Yeong W.Y. (2022). Investigating the effectiveness of three-dimensionally printed anatomical models compared with plastinated human specimens in learning cardiac and neck anatomy: A randomized crossover study. Anat. Sci. Educ..

[103] Arango S., Gorbaty B., Brigham J., Iaizzo P.A., Perry T.E. (2023). A role for ultra-high resolution three-dimensional printed human heart models. Echocardiography.

[104] Valverde I., Gomez-Ciriza G., Hussain T., Suarez-Mejias C., Velasco-Forte M.N., Byrne N., Ordoñez A., Gonzalez-Calle A., Anderson D., Hazekamp M.G. (2017). Three-dimensional printed models for surgical planning of complex congenital heart defects: An international multicentre study. Eur. J. Cardiothorac. Surg..

[105] Cen J., Liufu R., Wen S., Qiu H., Liu X., Chen X., Yuan H., Huang M., Zhuang J. (2021). Three-Dimensional Printing, Virtual Reality and Mixed Reality for Pulmonary Atresia: Early Surgical Outcomes Evaluation. Heart Lung. Circ..

[106] Guo H.C., Wang Y., Dai J., Ren C.W., Li J.H., Lai Y.Q. (2018). Application of 3D printing in the surgical planning of hypertrophic obstructive cardiomyopathy and physician-patient communication: A preliminary study. J. Thorac. Dis..

[107] Ryan J., Plasencia J., Richardson R., Velez D., Nigro J.J., Pophal S., Frakes D. (2018). 3D printing for congenital heart disease: A single site’s initial three-yearexperience. 3D Print. Med..

[108] Zhao L., Zhou S., Fan T., Li B., Liang W., Dong H. (2018). Three-dimensional printing enhances preparation for repair of double outlet right ventricular surgery. J. Cardiac. Surg..

[109] Ghosh R.M., Jolley M.A., Mascio C.E., Chen J.M., Fuller S., Rome J.J., Silvestro E., Whitehead K.K. (2022). Clinical 3D modeling to guide pediatric cardiothoracic surgery and intervention using 3D printed anatomic models, computer aided design and virtual reality. 3D Print. Med..

[110] Hell M.M., Achenbach S., Yoo I.S., Franke J., Blachutzik F., Roether J., Graf V., Raaz-Schrauder D., Marwan M., Schlundt C. (2017). 3D printing for sizing left atrial appendage closure device: Head-to-head comparison with computed tomography and transoesophageal echocardiography. EuroIntervention.

[111] Russo J.J., Yuen T., Tan J., Willson A.B., Gurvitch R. (2022). Assessment of Coronary Artery Obstruction Risk during Transcatheter Aortic Valve Replacement Utilising 3D-Printing. Heart Lung Circ..

[112] Fan Y., Yang F., Cheung G.S.-H., Chan A.K.-Y., Wang D.D., Lam Y.-Y., Chow M.C.-K., Leong M.C.-W., Kam K.K.-H., So K.C.-Y. (2019). Device Sizing Guided by Echocardiography-Based Three-Dimensional Printing Is Associated with Superior Outcome after Percutaneous Left Atrial Appendage Occlusion. J. Am. Soc. Echocardiogr..

[113] Wu C.-A., Squelch A., Jansen S., Sun Z. (2021). Optimization of computed tomography angiography protocols for follow-up type B aortic dissection patients by using 3D printed model. Appl. Sci..

[114] Xenofontos P., Zamani R., Akrami M. (2022). The application of 3D printing in preoperative planning for transcatheter aortic valve replacement: A systematic review. Biomed. Eng. Online.

[115] Tanaka Y., Saito S., Sasuga S., Takahashi A., Aoyama Y., Obama K., Umezu M., Iwasaki K. (2018). Quantitative assessment of paravalvular leakage after transcatheter aortic valve replacement using a patient-specific pulsatile flow model. Int. J. Cardiol..

[116] Brunner B.S., Thierij A., Jakob A., Tengler A., Grab M., Thierfelder N., Leuner C.J., Haas N.A., Hopfner C. (2022). 3D-printed heart models for hands-on training in pediatric cardiology—The future of modern learning and teaching?. GMS J. Med. Educ..

[117] Li H., Qingyao, Bingshen, Shu M., Lizhong, Wang X., Song Z. (2017). Application of 3D printing technology to left atrial appendage occlusion. Int. J. Cardiol..

[118] Conti M., Marconi S., Muscogiuri G., Guglielmo M., Baggiano A., Italiano G., Mancini M.E., Auricchio F., Andreini D., Rabbat M.G. (2019). Left atrial appendage closure guided by 3D computed tomography printing technology: A case control study. J. Cardiovasc. Comput. Tomogr..

[119] Goitein O., Fink N., Guetta V., Beinart R., Brodov Y., Konen E., Goitein D., Di Segni E., Grupper A., Glikson M. (2017). Printed MDCT 3D models for prediction of left atrial appendage (LAA) occluder device size: A feasibility study. EuroIntervention.

[120] Messarra B.T., Wang Y., Smith P.A., Peak P., Adams D.L., Crane T.N. (2023). 3D-Printed silicone anatomic patient simulator to enhance training on cardiopulmonary bypass. J. ExtraCorpor. Technol..

[121] Traynor G., Shearn A.I., Milano E.G., Ordonez M.V., Forte M.N.V., Caputo M., Schievano S., Mustard H., Wray J., Biglino G. (2022). The use of 3D-printed models in patient communication: A scoping review. J. 3D Print. Med..

[122] Illmann C.F., Hosking M., Harris K.C. (2020). Utility and Access to 3-Dimensional Printing in the Context of Congenital Heart Disease: An International Physician Survey Study. CJC Open..

[123] Biglino G., Capelli C., Leaver L.-K., Schievano S., Taylor A.M., Wray J. (2015). Involving patients, families and medical staff in the evaluation of 3D printing models of congenital heart disease. Commun. Med..

[124] Lau I.W.W., Liu D., Xu L., Fan Z., Sun Z. (2018). Clinical value of patient-specific three-dimensional printing of congenital heart disease: Quantitative and qualitative assessments. PLoS ONE.

[125] Biglino G., Koniordou D., Gasparini M., Capelli C., Leaver L.-K., Khambadkone S., Schievano S., Taylor A.M., Wray J. (2017). Piloting the Use of Patient-Specific Cardiac Models as a Novel Tool to Facilitate Communication During Cinical Consultations. Pediatr. Cardiol..

[126] Giovanni B., Claudio C., Jo W., Silvia S., Lindsay-Kay L., Sachin K., Alessandro G., Graham D., Alexander J., Andrew M.T. (2015). 3D-manufactured patient-specific models of congenital heart defects for communication in clinical practice: Feasibility and acceptability. BMJ Open.

[127] Biglino G., Moharem-Elgamal S., Lee M., Tulloh R., Caputo M. (2017). The Perception of a Three-Dimensional-Printed Heart Model from the Perspective of Different Stakeholders: A Complex Case of Truncus Arteriosus. Front. Pediatr..

[128] Deng X., He S., Huang P., Luo J., Yang G., Zhou B., Xiao Y. (2021). A three-dimensional printed model in preoperative consent for ventricular septal defect repair. J. Cardiothorac. Surg..

[129] Wu C.-A., Squelch A., Sun Z. (2021). Investigation of Three-dimensional Printing Materials for Printing Aorta Model Replicating Type B Aortic Dissection. Curr. Med. Imaging Rev..

[130] Sun Z., Ng C.K.C., Wong Y.H., Yeong C.H. (2021). 3D-Printed Coronary Plaques to Simulate High Calcification in the Coronary Arteries for Investigation of Blooming Artifacts. Biomolecules.

[131] Sun Z., Ng C.K.C., Squelch A. (2019). Synchrotron radiation computed tomography assessment of calcified plaques and coronary stenosis with different slice thicknesses and beam energies on 3D printed coronary models. Quant. Imaging Med. Surg..

[132] Sun Z. (2019). 3D printed coronary models offer new opportunities for developing optimal coronary CT angiography protocols in imaging coronary stents. Quant. Imaging Med. Surg..

[133] Sun Z. (2021). 3D printing in medical applications. Curr. Med. Imaging..

[134] Sommer K.N., Iyer V., Kumamaru K.K., Rava R.A., Ionita C.N. (2020). Method to simulate distal flow resistance in coronary arteries in 3D printed patient specific coronary models. 3D Print. Med..

[135] Wu C.-A., Squelch A., Sun Z. (2022). Assessment of optimization of computed tomography angiography protocols for follow-up type B aortic dissection patients by using a 3D-printed model. J. 3D Print. Med..

[136] Aldosari S., Jansen S., Sun Z. (2019). Optimization of computed tomography pulmonary angiography protocols using 3D printed model with simulation of pulmonary embolism. Quant. Imaging Med. Surg..

[137] Aldosari S., Jansen S., Sun Z. (2019). Patient-specific 3D printed pulmonary artery model with simulation of peripheral pulmonary embolism for developing optimal computed tomography pulmonary angiography protocols. Quant. Imaging Med. Surg..

[138] Sun Z., Jansen S. (2019). Personalized 3D printed coronary models in coronary stenting. Quant. Imaging Med. Surg..

[139] Abdullah K.A., McEntee M.F., Reed W., Kench P.L. (2018). Development of an organ-specific insert phantom generated using a 3D printer for investigations of cardiac computed tomography protocols. J. Med. Radiat. Sci..

[140] Mørup S.D., Stowe J., Precht H., Gervig M.H., Foley S. (2022). Design of a 3D printed coronary artery model for CT optimization. Radiography.

[141] Ripley B., Kelil T., Cheezum M.K., Goncalves A., Di Carli M.F., Rybicki F.J., Steigner M., Mitsouras D., Blankstein R. (2016). 3D printing based on cardiac CT assists anatomic visualization prior to transcatheter aortic valve replacement. J. Cardiovasc. Comput. Tomogr..

[142] Kiraly L., Shah N.C., Abdullah O., Al-Ketan O., Rowshan R. (2021). Three-Dimensional Virtual and Printed Prototypes in Complex Congenital and Pediatric Cardiac Surgery—A Multidisciplinary Team-Learning Experience. Biomolecules.

[143] Ullah M., Bibi A., Wahab A., Hamayun S., Rehman M.U., Khan S.U., Awan U.A., Riaz N.-u.-a., Naeem M., Saeed S. (2024). Shaping the Future of Cardiovascular Disease by 3D Printing Applications in Stent Technology and its Clinical Outcomes. Curr. Probl. Cardiol..

[144] Sun Z., Zhao J., Leung E., Flandes-Iparraguirre M., Vernon M., Silberstein J., De-Juan-Pardo E.M., Jansen S. (2023). Three-Dimensional Bioprinting in Cardiovascular Disease: Current Status and Future Directions. Biomolecules.

[145] Jana S., Tefft B.J., Spoon D.B., Simari R.D. (2014). Scaffolds for tissue engineering of cardiac valves. Acta Biomater..

[146] Mela P. (2020). Subject- and Leaflet-Specific Remodeling of Polymeric Heart Valves for In Situ Tissue Engineering. JACC Basic. Transl. Sci..

[147] Vesely I. (2005). Heart Valve Tissue Engineering. Circ. Res..

[148] Wissing T.B., Bonito V., Bouten C.V.C., Smits A.I.P.M. (2017). Biomaterial-driven in situ cardiovascular tissue engineering—A multi-disciplinary perspective. npj Regen. Med..

[149] Butcher J.T. (2018). The root problem of heart valve engineering. Sci. Transl. Med..

[150] Tomasina C., Bodet T., Mota C., Moroni L., Camarero-Espinosa S. (2019). Bioprinting Vasculature: Materials, Cells and Emergent Techniques. Materials.

[151] Seymour A.J., Westerfield A.D., Cornelius V.C., Skylar-Scott M.A., Heilshorn S.C. (2022). Bioprinted microvasculature: Progressing from structure to function. Biofabrication.

[152] Wang Z., Wang L., Li T., Liu S., Guo B., Huang W., Wu Y. (2021). 3D bioprinting in cardiac tissue engineering. Theranostics.

[153] Bejleri D., Streeter B.W., Nachlas A.L.Y., Brown M.E., Gaetani R., Christman K.L., Davis M.E. (2018). A Bioprinted Cardiac Patch Composed of Cardiac-Specific Extracellular Matrix and Progenitor Cells for Heart Repair. Adv. Healthc. Mater..

[154] Zhu K., Shin S.R., van Kempen T., Li Y.C., Ponraj V., Nasajpour A., Mandla S., Hu N., Liu X., Leijten J. (2017). Tissue Engineering: Gold Nanocomposite Bioink for Printing 3D Cardiac Constructs. Adv. Funct. Mater..

[155] Erdem A., Darabi M.A., Nasiri R., Sangabathuni S., Ertas Y.N., Alem H., Hosseini V., Shamloo A., Nasr A.S., Ahadian S. (2020). 3D Bioprinting of Oxygenated Cell-Laden Gelatin Methacryloyl Constructs. Adv. Healthc. Mater..

[156] Ahrens J.H., Uzel S.G.M., Skylar-Scott M., Mata M.M., Lu A., Kroll K.T., Lewis J.A. (2022). Programming Cellular Alignment in Engineered Cardiac Tissue via Bioprinting Anisotropic Organ Building Blocks. Adv. Mater..

[157] Asulin M., Michael I., Shapira A., Dvir T. (2021). One-Step 3D Printing of Heart Patches with Built-in Electronics for Performance Regulation. Adv. Sci..

[158] Häneke T., Sahara M. (2022). Progress in Bioengineering Strategies for Heart Regenerative Medicine. Int. J. Mol. Sci..

[159] Zhou Z., Tang W., Yang J., Fan C. (2023). Application of 4D printing and bioprinting in cardiovascular tissue engineering. Biomater. Sci..

[160] Chessa M., Van De Bruaene A., Farooqi K., Valverde I., Jung C., Votta E., Sturla F., Diller G.P., Brida M., Sun Z. (2022). Three-dimensional printing, holograms, computational modelling, and artificial intelligence for adult congenital heart disease care: An exciting future. Eur. Heart J..

[161] Sun Z., Wee C. (2022). 3D Printed Models in Cardiovascular Disease: An Exciting Future to Deliver Personalized Medicine. Micromachines.

[162] Gharleghi R., Desalles C.A., Lal R., McCraith S., Sarathy K., Jepson N., Otton J., Barakat A.I., Beier S. (2021). 3D printing for cardiovascular applications: From end-to-end processes to emerging developments. Ann. Biomed. Eng..

[163] Pijls N.H.J., Van Schaardenburgh P., De Bruyne B., Manoharan G., Boersma E., Bech J.-W., Vant Veer M., BÄR F., Hoorntje J., Koolen J. (2007). Percutaneous coronary intervention of functionally nonsignificant stenosis: 5-year follow-up of the DEFER Study. J. Am. Coll. Cardiol..

[164] Zimmermann F.M., Ferrara A., Johnson N.P., van Nunen L.X., Escaned J., Albertsson P., Erbel R., Legrand V., Gwon H.-C., Remkes W.S. (2015). Deferral vs. performance of percutaneous coronary intervention of functionally non-significant coronary stenosis: 15-year follow-up of the DEFER trial. Eur. Heart J..

[165] Tonino P.A.L., De Bruyne B., Pijls N.H.J., Siebert U., Ikeno F., van’t Veer M., Klauss V., Manoharan G., Engstrøm T., Oldroyd K.G. (2009). Fractional Flow Reserve versus Angiography for Guiding Percutaneous Coronary Intervention. N. Eng. J. Med..

[166] Pijls N.H.J., Fearon W.F., Oldroyd K.G., Ver Lee P.N., Maccarthy P.A., De Bruyne B., Tonino P.A.L., Siebert U., Ikeno F., Bornschein B. (2010). Fractional Flow Reserve Versus Angiography for Guiding Percutaneous Coronary Intervention in Patients with Multivessel Coronary Artery Disease: 2-Year Follow-up of the FAME (Fractional Flow Reserve Versus Angiography for Multivessel Evaluation) Study. J. Am. Coll. Cardiol..

[167] Van Nunen L.X.M.D., Zimmermann F.M.M.D., Tonino P.A.L.P., Barbato E.P., Baumbach A.P., Engstrøm T.P., Klauss V.P., MacCarthy P.A.P., Manoharan G.M.D., Oldroyd K.G.P. (2015). Fractional flow reserve versus angiography for guidance of PCI in patients with multivessel coronary artery disease (FAME): 5-year follow-up of a randomised controlled trial. Lancet.

[168] Fearon W.F., Tonino P.A., De Bruyne B., Siebert U., Pijls N.H. (2007). Rationale and design of the fractional flow reserve versus angiography for multivessel evaluation (FAME) study. Am. Heart J..

[169] Fearon W.F., Bornschein B., Tonino P.A.L., Gothe R.M., De Bruyne B., Pijls N.H.J., Siebert U. (2010). Economic Evaluation of Fractional Flow Reserve-Guided Percutaneous Coronary Intervention in Patients With Multivessel Disease. Circulation.

[170] De Bruyne B., Pijls N.H.J., Kalesan B., Barbato E., Tonino P.A.L., Piroth Z., Jagic N., Mobius-Winckler S., Rioufol G., Witt N. (2012). Fractional Flow Reserve–Guided PCI versus Medical Therapy in Stable Coronary Disease. N. Eng. J. Med..

[171] Maznyczka A.M., Matthews C.J., Blaxill J.M., Greenwood J.P., Mozid A.M., Rossington J.A., Veerasamy M., Wheatcroft S.B., Curzen N., Bulluck H. (2022). Fractional Flow Reserve versus Angiography-Guided Management of Coronary Artery Disease: A Meta-Analysis of Contemporary Randomised Controlled Trials. J. Clin. Med..

[172] Yang J., Shan D., Wang X., Sun X., Shao M., Wang K., Pan Y., Wang Z., Schoepf U.J., Savage R.H. (2023). On-Site Computed Tomography-Derived Fractional Flow Reserve to Guide Management of Patients with Stable Coronary Artery Disease: The TARGET Randomized Trial. Circulation.

[173] Jung C., Wolff G., Wernly B., Bruno R.R., Franz M., Schulze P.C., Silva J.N.A., Silva J.R., Bhatt D.L., Kelm M. (2022). Virtual and Augmented Reality in Cardiovascular Care: State-of-the-Art and Future Perspectives. JACC Cardiovasc. Imaging.

[174] Mitsuno D., Ueda K., Hirota Y., Ogino M. (2019). Effective Application of Mixed Reality Device HoloLens: Simple Manual Alignment of Surgical Field and Holograms. Plast. Reconstr. Surg..

[175] Moro C., Phelps C., Redmond P., Stromberga Z. (2021). HoloLens and mobile augmented reality in medical and health science education: A randomised controlled trial. Br. J. Educ. Technol..

[176] Gehrsitz P., Rompel O., Schöber M., Cesnjevar R., Purbojo A., Uder M., Dittrich S., Alkassar M. (2021). Cinematic Rendering in Mixed-Reality Holograms: A New 3D Preoperative Planning Tool in Pediatric Heart Surgery. Front. Cardiovasc. Med..

[177] Soulami R.B., Verhoye J.-P., Duc H.N., Castro M., Auffret V., Anselmi A., Haigron P., Ruggieri V.G. (2016). Computer-Assisted Transcatheter Heart Valve Implantation in Valve-in-Valve Procedures. Innovations.

[178] Opolski M.P., Debski A., Borucki B.A., Staruch A.D., Kepka C., Rokicki J.K., Sieradzki B., Witkowski A. (2017). Feasibility and safety of augmented-reality glass for computed tomography-assisted percutaneous revascularization of coronary chronic total occlusion: A single center prospective pilot study. J. Cardiovasc. Comput. Tomogr..

[179] Ye W., Zhang X., Li T., Luo C., Yang L. (2021). Mixed-reality hologram for diagnosis and surgical planning of double outlet of the right ventricle: A pilot study. Clin. Radiol..

[180] Kumar R.P., Pelanis E., Bugge R., Brun H., Palomar R., Aghayan D.L., Fretland Å.A., Edwin B., Elle O.J. (2020). Use of mixed reality for surgery planning: Assessment and development workflow. J. Biomed. Inform..

[181] Brun H., Bugge R.A.B., Suther L.K.R., Birkeland S., Kumar R., Pelanis E., Elle O.J. (2019). Mixed reality holograms for heart surgery planning: First user experience in congenital heart disease. Eur. Heart J. Cardiovasc. Imaging.

[182] Lau I., Gupta A., Sun Z. (2021). Clinical Value of Virtual Reality versus 3D Printing in Congenital Heart Disease. Biomolecules.

[183] Lau I., Gupta A., Ihdayhid A., Sun Z. (2022). Clinical Applications of Mixed Reality and 3D Printing in Congenital Heart Disease. Biomolecules.

[184] Kim B., Loke Y.-H., Mass P., Irwin M.R., Capeland C., Olivieri L., Krieger A. (2020). A Novel Virtual Reality Medical Image Display System for Group Discussions of Congenital Heart Disease: Development and Usability Testing. JMIR Cardiol..

[185] Patel N., Costa A., Sanders S.P., Ezon D. (2021). Stereoscopic virtual reality does not improve knowledge acquisition of congenital heart disease. Int. J. Cardiovasc. Imaging.

[186] Rad A.A., Vardanyan R., Thavarajasingam S.G., Zubarevich A., Eynde J.V.D., Sá M.P.B.O., Zhigalov K., Nia P.S., Ruhparwar A., Weymann A. (2022). Extended, virtual and augmented reality in thoracic surgery: A systematic review. Interact. Cardiovasc. Thorac. Surg..

[187] Wang H., Wang R., Li Y., Zhou Z., Gao Y., Bo K., Yu M., Sun Z., Xu L. (2022). Assessment of Image Quality of Coronary Computed Tomography Angiography in Obese Patients by Comparing Deep Learning Image Reconstruction with Adaptive Statistical Iterative Reconstruction Veo. J. Comput. Assist. Tomogr..

[188] Wang W., Wang H., Chen Q., Zhou Z., Wang R., Wang H., Zhang N., Chen Y., Sun Z., Xu L. (2020). Coronary artery calcium score quantification using a deep-learning algorithm. Clin. Radiol..

[189] Han D., Liu J., Sun Z., Cui Y., He Y., Yang Z. (2020). Deep learning analysis in coronary computed tomographic angiography imaging for the assessment of patients with coronary artery stenosis. Comput. Methods Programs. Biomed..

[190] Gao Y., Wang W., Wang H., Zhou Z., Xu P., Jiang M., Yang L., Wang H., Wen H., Sun Z. (2022). Impact of Sublingual Nitroglycerin on the Assessment of Computed Tomography–derived Fractional Flow Reserve: An Intraindividual Comparison Study. J. Comput. Assist. Tomogr..

[191] Mu D., Bai J., Chen W., Yu H., Liang J., Yin K., Li H., Qing Z., He K., Yang H.Y. (2022). Calcium scoring at coronary CT angiography using deep learning. Radiology.

[192] Li P., Xu L., Yang L., Wang R., Hsieh J., Sun Z., Fan Z., Leipsic J.A. (2018). Blooming Artifact Reduction in Coronary Artery Calcification by a New De-blooming Algorithm: Initial Study. Sci. Rep..

[193] Alskaf E., Dutta U., Scannell C.M., Chiribiri A. (2022). Deep learning applications in coronary anatomy imaging: A systematic review and meta-analysis. J. Med. Artif. Intell..

[194] Lin A., Manral N., McElhinney P., Killekar A., Matsumoto H., Kwiecinski J., Pieszko K., Razipour A., Grodecki K., Park C. (2022). Deep learning-enabled coronary CT angiography for plaque and stenosis quantification and cardiac risk prediction: An international multicentre study. Lancet.

[195] Jávorszky N., Homonnay B., Gerstenblith G., Bluemke D., Kiss P., Török M., Celentano D., Lai H., Lai S., Kolossváry M. (2022). Deep learning–based atherosclerotic coronary plaque segmentation on coronary CT angiography. Eur. Radiol..

[196] Dey D., Slomka P.J., Leeson P., Comaniciu D., Shrestha S., Sengupta P.P., Marwick T.H. (2019). Artificial Intelligence in Cardiovascular Imaging: JACC State-of-the-Art Review. J. Am. Coll. Cardiol..

[197] Zreik M., van Hamersvelt R.W., Wolterink J.M., Leiner T., Viergever M.A., Isgum I. (2019). A Recurrent CNN for Automatic Detection and Classification of Coronary Artery Plaque and Stenosis in Coronary CT Angiography. IEEE. Trans. Med. Imaging.

[198] Choi A.D., Marques H., Kumar V., Griffin W.F., Rahban H., Karlsberg R.P., Zeman R.K., Katz R.J., Earls J.P. (2021). CT Evaluation by Artificial Intelligence for Atherosclerosis, Stenosis and Vascular Morphology (CLARIFY): A Multi-center, international study. J. Cardiovasc. Comput. Tomogr..

[199] Kang D., Dey D., Slomka P.J., Arsanjani R., Nakazato R., Ko H., Berman D.S., Li D., Kuo C.C.J. (2015). Structured learning algorithm for detection of nonobstructive and obstructive coronary plaque lesions from computed tomography angiography. J. Med. Imaging.

[200] Raffort J., Adam C., Carrier M., Ballaith A., Coscas R., Jean-Baptiste E., Hassen-Khodja R., Chakfé N., Lareyre F. (2020). Artificial intelligence in abdominal aortic aneurysm. J. Vasc. Surg..

[201] Lareyre F., Adam C., Carrier M., Dommerc C., Mialhe C., Raffort J. (2019). A fully automated pipeline for mining abdominal aortic aneurysm using image segmentation. Sci. Rep..

[202] Spinella G., Fantazzini A., Finotello A., Vincenzi E., Boschetti G.A., Brutti F., Magliocco M., Pane B., Basso C., Conti M. (2023). Artificial Intelligence Application to Screen Abdominal Aortic Aneurysm Using Computed tomography Angiography. J. Digit. Imaging.

[203] Attallah O., Karthikesalingam A., Holt P.J., Thompson M.M., Sayers R., Bown M.J., Choke E.C., Ma X. (2017). Using multiple classifiers for predicting the risk of endovascular aortic aneurysm repair re-intervention through hybrid feature selection. Proc. Inst. Mech. Eng. Part H.

[204] Wise E.S., Hocking K.M., Brophy C.M. (2015). Prediction of in-hospital mortality after ruptured abdominal aortic aneurysm repair using an artificial neural network. J. Vasc. Surg..

[205] Lee R., Jarchi D., Perera R., Jones A., Cassimjee I., Handa A., Clifton D.A., Bellamkonda K., Woodgate F., Killough N. (2018). Applied Machine Learning for the Prediction of Growth of Abdominal Aortic Aneurysm in Humans. EJVES Short Rep..

[206] Hata A., Yanagawa M., Yamagata K., Suzuki Y., Kido S., Kawata A., Doi S., Yoshida Y., Miyata T., Tsubamoto M. (2021). Deep learning algorithm for detection of aortic dissection on non-contrast-enhanced CT. Eur. Radiol..

[207] Zhou Z., Gao Y., Zhang W., Bo K., Zhang N., Wang H., Wang R., Du Z., Firmin D., Yang G. (2023). Artificial intelligence–based full aortic CT angiography imaging with ultra-low-dose contrast medium: A preliminary study. Eur. Radiol..

[208] Chandra S., Sarkar P.K., Chandra D., Ginsberg N.E., Cohen R.I. (2013). Finding an alternative diagnosis does not justify increased use of CT-pulmonary angiography. BMC Pulm. Med..

[209] Soffer S., Klang E., Shimon O., Barash Y., Cahan N., Greenspana H., Konen E. (2021). Deep learning for pulmonary embolism detection on computed tomography pulmonary angiogram: A systematic review and meta-analysis. Sci. Rep..

[210] Huhtanen H., Nyman M., Mohsen T., Virkki A., Karlsson A., Hirvonen J. (2022). Automated detection of pulmonary embolism from CT-angiograms using deep learning. BMC Med. Imaging.

[211] Ma X., Ferguson E.C., Jiang X., Savitz S.I., Shams S. (2022). A multitask deep learning approach for pulmonary embolism detection and identification. Sci. Rep..

[212] Grenier P.A., Ayobi A., Quenet S., Tassy M., Marx M., Chow D.S., Weinberg B.D., Chang P.D., Chaibi Y. (2023). Deep Learning-Based Algorithm for Automatic Detection of Pulmonary Embolism in Chest CT Angiograms. Diagnostics.

[213] Colak E., Kitamura F.C., Hobbs S.B., Wu C.C., Lungren M.P., Prevedello L.M., Kalpathy-Cramer J., Ball R.L., Shih G., Stein A. (2021). The RSNA Pulmonary Embolism CT Dataset. Radiol. Artif. Intell..

[214] Zhang N., Zhao X., Li J., Huang L., Li H., Feng H., Garcia M.A., Cao Y., Sun Z., Chai S. (2023). Machine Learning Based on Computed Tomography Pulmonary Angiography in Evaluating Pulmonary Artery Pressure in Patients with Pulmonary Hypertension. J. Clin. Med..

[215] Sun Z. (2023). Patient-Specific 3D-Printed Models in Pediatric Congenital Heart Disease. Children.

[216] Jiang B., Guo N., Ge Y., Zhang L., Oudkerk M., Xie X. (2020). Development and application of artificial intelligence in cardiac imaging. Br. J. Radiol..

